# Quartz-enhanced laser spectroscopy sensing

**DOI:** 10.1038/s41377-025-02075-7

**Published:** 2026-01-01

**Authors:** Shunda Qiao, Xiaonan Liu, Ziting Lang, Ying He, Weidong Chen, Yufei Ma

**Affiliations:** 1 National Key Laboratory of Laser Spatial Information, Harbin Institute of Technology, Harbin, 150000 China; 2 Zhengzhou Research Institute, Harbin Institute of Technology, Zhengzhou, 450000 China; 3 Laboratoire de Physicochimie de l’Atmosphère, Université du Littoral Côte d’Opale, Dunkerque, 59140 France

**Keywords:** Optical spectroscopy, Optical metrology

## Abstract

Gas sensing technology is widely applied in various fields, including environmental monitoring, industrial process control, medical diagnostics, safety warnings, and more. As a detection element, the quartz tuning fork (QTF) offers advantages such as high-quality factor (Q-factor), strong noise immunity, compact size, and low cost. Notably, its resonant characteristics significantly enhance system signal strength. Two spectroscopic techniques based on QTF detection, Quartz-enhanced photoacoustic spectroscopy (QEPAS) and light-induced thermoelastic spectroscopy (LITES), are currently research hotspots in the field of spectral sensing. This paper provides a comprehensive and detailed review and highlights pivotal innovations in these two QTF-based spectroscopic techniques. For QEPAS, these encompass high-power excitation methods, novel excitation sources, advanced QTF detection elements, and acoustic wave amplification strategies. Regarding LITES, the researches on optical cavity-enhanced approaches, modified QTF improvement mechanisms, integration with heterodyne demodulation technique, and combination with QEPAS were analyzed. These advances have enabled quartz-enhanced laser spectroscopy to achieve detection limits ranging from parts-per-billion (ppb) to parts-per-trillion (ppt) levels for trace gases such as methane (CH₄), acetylene (C₂H₂), carbon monoxide (CO), and so on. Additionally, prospects for future technological developments are also discussed in the concluding section.

## Abstract (web summary)

QEPAS and LITES, collectively called quartz-enhanced laser spectroscopy technology, may bring a paradigm shift in gas sensing for applications.

## Graphical abstract

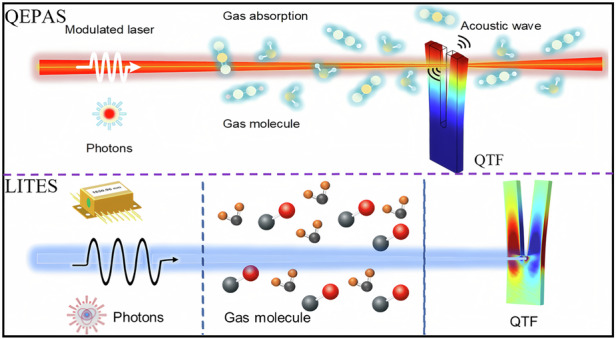

## Introduction

**Fig. 1:**
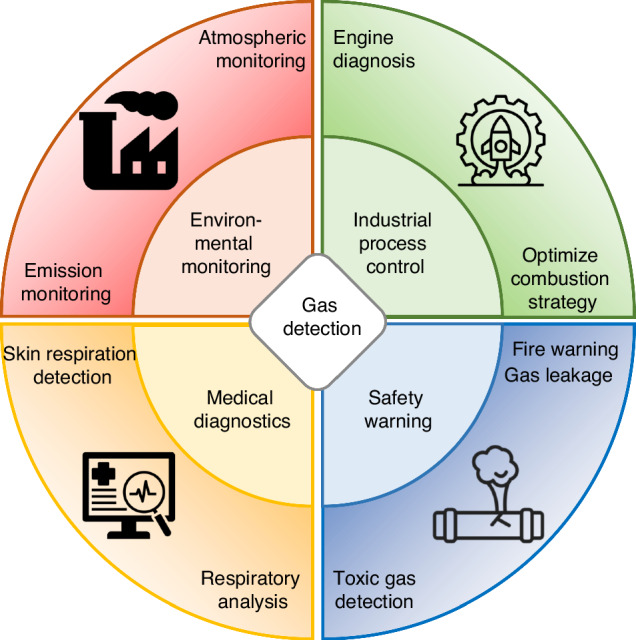
Applications of gas detection technology

Gas sensing technology plays a crucial role in various fields such as environmental monitoring, industrial process control, medical diagnostics, and safety warnings^1–4^, as shown in Fig. 1. With the acceleration of industrialization and the increasing severity of environmental pollution issues, the demand for highly sensitive and selective detection technologies for trace gases is continuously growing. Although gas sensing has been widely researched, high-sensitivity and fast response trace gas detection still faces enormous challenges^5–9^. Electrochemical sensors and semiconductor sensors, traditionally used for gas detection, are recognized for their cost-effectiveness and ease of integration. However, in contrast, spectral gas sensors show significant advantages in sensitivity, selectivity, and resilience to interferences, gradually becoming a more reliable alternative.

Spectroscopic sensing technologies leverage the absorption^10,11^, emission^12,13^, or scattering of specific substances^14–19^ to ascertain the type and concentration of gases. Absorption spectroscopy, capitalizing on its unique “fingerprint” characteristics, is highly esteemed in gas detection for its high sensitivity and selectivity^20–23^. It is based on Lambert-Beer’s law, which describes the relationship between the substance’s absorption of light and gas concentration along the optical path^24–27^. Most absorption spectroscopy techniques, such as tunable diode laser absorption spectroscopy and differential optical absorption spectroscopy, rely on photodetectors to detect spectral signals^28,29^. However, photodetectors have limitations in their response wavelength range, and they cannot detect lasers with wavelengths longer than 10 μm. In addition, the high cost and the need for cooling of mid-infrared photodetectors further restrict their applications^30^. Photoacoustic spectroscopy (PAS), detected using a microphone, is based on the photoacoustic effect, which occurs when light interacts with matter^31^. It is widely used due to its unique “zero-background” feature^32–37^. In 2002, Kosterev et al. introduced quartz-enhanced photoacoustic spectroscopy (QEPAS) technology, replacing the traditional microphone in PAS with a quartz tuning fork (QTF) as the detecting element^36^. The QTF, a miniature resonator with piezoelectric effect, is characterized by a high-quality factor (>10,000 at atmospheric pressure), excellent frequency stability, and high sensitivity^37–41^. Additionally, its compact size (<0.3 mm^2^), lightweight, and no power consumption demonstrate promising applications in portable and miniaturized gas sensors. However, due to its contact detection characteristic, QEPAS cannot be applied to the detection of corrosive gases or combustion field^42,43^. In 2018, Ma et al. proposed light-induced thermoelastic spectroscopy (LITES) based on the light-induced thermoelastic effect of QTF^44,45^. This technology not only inherits the advantages of QEPAS but also achieves non-contact detection. The broad spectral response band from ultraviolet to terahertz wave effectively expands the detection band of laser absorption spectroscopy sensing technology^46^, thereby enhancing the performance and efficiency of the sensing system.

In summary, although various techniques exist in the field of gas sensing, this review focuses on QEPAS and LITES primarily due to their unique innovations: both utilize a miniature QTF as the core detection element, converting light energy absorbed by the gas into a highly sensitive vibration signal of the QTF. QEPAS directly detects acoustic wave resonance via the QTF, while LITES measures the QTF vibration excited by laser-induced thermal expansion, eliminating the need for traditional optical detectors. Since the QTF possesses the characteristics of small size, low-cost, high-quality factor, and wavelength independence, this confers three key advantages upon quartz-enhanced laser spectroscopy sensing technology: (1) Excellent environmental noise immunity. (2) Potential for chip-scale miniaturization. (3) Ultra-high sensitivity from ppm to ppt levels. In contrast, other techniques, due to limitations in size or detector performance, struggle to meet the urgent demand for current portable, high-precision sensing. Therefore, given the boundless development potential of quartz-enhanced laser spectroscopy, this paper specifically explores these two QTF detection-based technologies, which were current research hotspots in the gas sensing field. Their breakthrough progress is expected to drive a paradigm shift in gas sensing for fields such as environmental monitoring and medical diagnostics.

Specifically, this review summarizes recent advances in two QTF-based gas sensing technologies of QEPAS and LITES. The review is divided into two parts: In the first part, the basic principle of QEPAS is explained, and recent research progress aimed at enhancing the detection performance of QEPAS sensors from various perspectives is discussed. In the second part, the principles of LITES technology and recent advancements in improving detection sensitivity and stability of LITES sensors are introduced.

## QEPAS-based gas sensing

### The principle of QEPAS

**Fig. 2:**
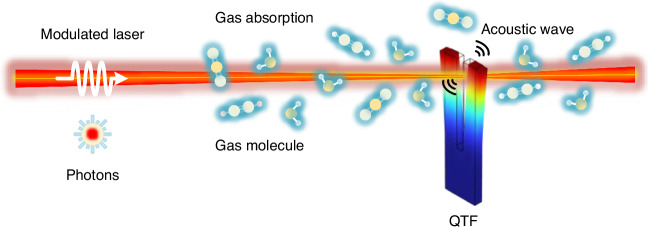
The principle of QEPAS technology

As an advancement of traditional microphone-based PAS, QEPAS operates on the fundamental principles of the photoacoustic effect^47–50^, as illustrated in Fig. 2. In QEPAS, gas molecules absorb modulated light of a specific wavelength and undergo non-radiative transitions from an excited state to the ground state. The absorbed light energy is converted into heat, causing a temperature change in the surrounding gas, which leads to periodic pressure variations. As a result, an acoustic wave is generated, with its frequency corresponding to the modulation frequency of the light. The amplitude of the acoustic wave is directly proportional to the gas concentration. Thus, an acoustic detector can be used to measure this amplitude and determine the gas concentration. In QEPAS, a QTF serves as the acoustic transducer with a sharp resonance. When the resonant frequency of the QTF matches the frequency of the generated acoustic wave, resonance occurs, amplifying the vibration of the QTF. Due to the piezoelectric effect, the vibration of the QTF prongs is converted into an electrical current. By measuring this electrical current, the gas concentration can be determined.

### Standard QEPAS sensor

**Fig. 3:**
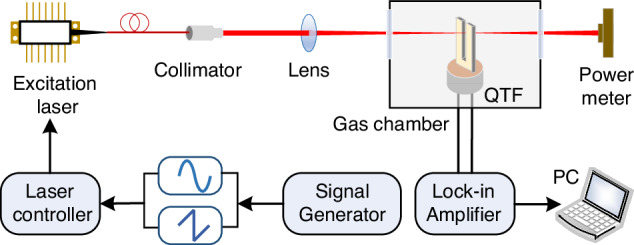
The standard QEPAS system configuration

Due to the attributes of QTF, such as its narrow bandwidth, high-quality factor (*Q*-factor), small size, and low cost, the QEPAS system offers superior advantages, including enhanced noise resistance, heightened detection performance, more compact structure, and more favorable economic benefits^47–51^. The configuration of the standard QEPAS sensor system consists of four main components: modulation signal production, gas absorption, acoustic wave detection, and data processing, as shown in Fig. 3.

In the optical path, after passing through a collimator and a focusing lens, the laser beam passes through the gap of the QTF. This arrangement ensures full interaction between the laser energy and gas molecules, thereby driving the QTF to generate the most potent fundamental frequency vibration. There are two methods for modulating laser to generate photoacoustic effect: intensity modulation and wavelength modulation spectroscopy (WMS)^52–54^. WMS can effectively mitigate system signal errors caused by laser wavelength drift as it tunes the laser wavelength. Furthermore, WMS completes the detection of gas absorption at high frequencies with an improved signal-to-noise ratio (SNR), thereby reducing noise. Consequently, spectral absorption information can be extracted through the analysis of various harmonic signals. Since the peak value of harmonic signals decreases with increasing harmonic order, and the highest peak in even harmonic signals occurs at the peak of the gas absorption line, second harmonic frequency (2*f*) analysis is commonly employed for most gas analysis applications^55–58^. To extract and analyze high-frequency signals at known carrier frequencies, a lock-in amplifier (LIA) is used to create bandpass filters at a specified frequency. If the bandwidth is sufficiently narrow, it can suppress broadband noise, achieving highly sensitive detection. The signal amplitude of the QEPAS system can be represented by Formula 1^50,59,60^.

1 $$S \sim \frac{\alpha PQ}{{f}_{0}}$$

where *f*_*0*_ is the resonant frequency of QTF, *α* is the absorption coefficient of the gas absorption line, *P* is the laser power, and *Q* is the *Q*-factor of QTF. According to Formula 1, the detection performance of the QEPAS sensor can be improved by optimizing the physical parameters mentioned above.

**Fig. 4:**
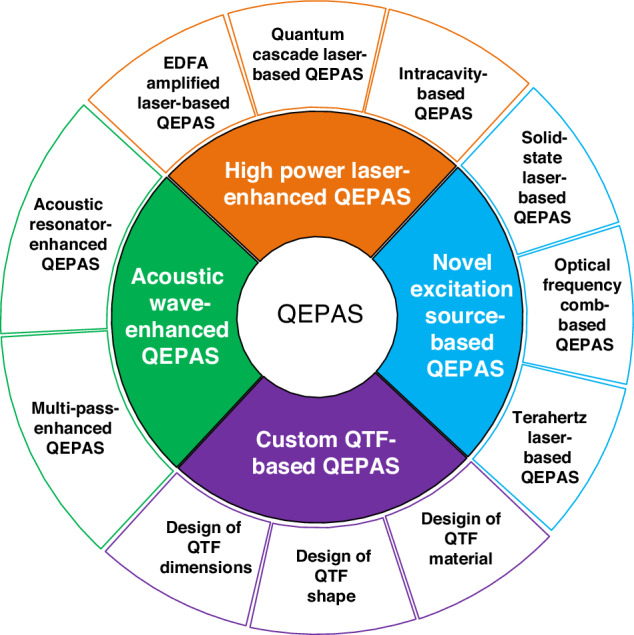
A summary of various approaches for improving QEPAS technology

Currently, there are several approaches to enhance the performance of QEPAS systems: (1) using high-power laser to actuate more gas molecules achieve absorption; (2) applying novel excitation source with strong absorption coefficient to strengthen the absorption; (3) utilizing a custom QTF to increase detected signal; (4) employing acoustic resonator to amplify acoustic wave or adopting multi-pass structure to generate multiple acoustic wave source. The various configurations of state-of-the-art QEPAS systems are summarized in Fig. 4. A detailed overview of recent advancements of the QEPAS technique is presented in the following.

### High-power laser-enhanced QEPAS sensor

The generation of acoustic waves in QEPAS relies on the absorption of light energy by gas molecules. According to the theory of gas infrared absorption spectroscopy, an increase in laser power can excite more gas molecules, leading to stronger non-radiative transitions and more powerful acoustic waves. Therefore, enhancing the excitation source power is an effective way to increase the QEPAS signal. Currently, near-infrared diode lasers are considered excellent excitation sources in QEPAS technology due to their compact size and fiber-coupled configuration^61–64^. However, the output power of near-infrared diode lasers is relatively low, typically ranging from a few milliwatts to tens of milliwatts, which limits the detection sensitivity of the QEPAS system. To achieve better detection performance, the main strategies currently employed include increasing the output power of the excitation source by using optical amplifiers, implementing intracavity structures, or directly using high-power excitation sources such as quantum cascade lasers (QCLs)^65–85^.

#### EDFA-amplified laser-based QEPAS sensor

**Fig. 5:**
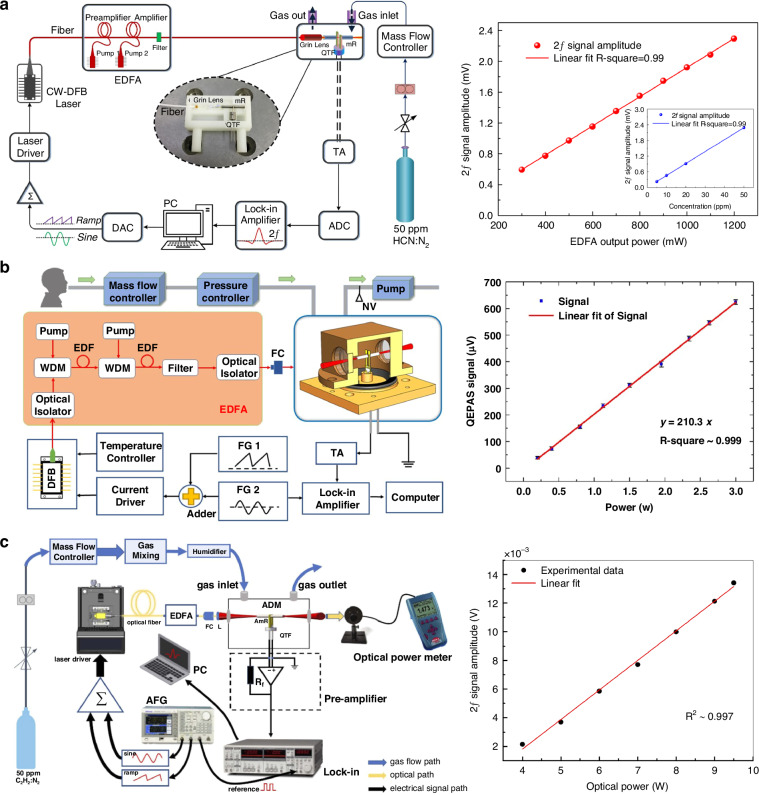
**Schematic of EDFA-amplified diode laser-based QEPAS sensors**. **a** A diode laser with an amplified output power of 1.2 W for HCN detection^66^. **b** A diode laser with an amplified output power of 3 W for NH_3_ detection^70^. **c** A diode laser with an amplified output power of 10 W for C_2_H_2_ detection^71^. **a** is reprinted from ref. ^66^ with permission from the Optica Publishing Group. **b** is reproduced from ref. ^70^ with permission from Elsevier. **c** is reprinted from ref. ^71^ with permission from the Optica Publishing Group

Erbium-doped fiber amplifier (EDFA) has been validated to be an effective method for optical amplification, offering several significant advantages, such as fiber compatibility, low loss, and high gain. Integrating an EDFA into the QEPAS technique effectively addresses the problem of low output power in near-infrared diode lasers, overcoming power limitations and enabling more sensitive gas detection. This has led to the development of EDFA-amplified laser-based QEPAS. This approach has been successfully demonstrated for the sensitive detection of ammonia (NH_3_), hydrogen sulfide (H_2_S), acetylene (C_2_H_2_), and hydrogen cyanide (HCN)^65–71^. The schematic configuration of EDFA-amplified laser-based QEPAS sensors is shown in Fig. 5, and the parameters and performance of these sensors are summarized in Table 1. A distributed feedback laser (DFB) diode laser generally serves as the excitation source. The output power of this diode laser can be boosted to hundreds of milliwatts or even several watts using an EDFA, resulting in a three-order-of-magnitude improvement. After being amplified by the EDFA, the output laser is incident on an acoustic detection module (ADM). As a result of that, ADM is filled with target gas, and the photoacoustic effect is produced. With the power amplifying, the performance of the QEPAS sensor is raised, and the minimum detection limit (MDL) for the EDFA-based QEPAS sensor was achieved at the ppb (parts per billion) or ppt (parts per trillion) level.

**Table 1:** The summary of the EDFA-amplified laser-based QEPAS sensors

Target gas	Amplified output power	Output wavelength	MDL @ Integration time	NNEA (cm^−1^ × W × Hz^−1/2^)
C_2_H_2_^65^	1.5 W	1.53 μm	33.2 ppb @ 1 s	3.5 × 10^−8^
HCN^66^	1.2 W	1.53 μm	220.0 ppt @ 300 s	1.1 × 10^−8^
H_2_S^67^	1.4 W	1.58 μm	734.0 ppb @ 1 s 142.0 ppb @ 67 s	9.8 × 10^−9^
H_2_S/NH_3_^68^	1.3 W	1.58 μm	17.0 ppb @ 132 s 52.0 ppb @ 132 s	1.4 × 10^−9^ 1.5 × 10^−9^
NH_3_^69^	1.0 W	1.53 μm	418.4 ppb @ 1 s	3.8 × 10^−8^
NH_3_^70^	3.0 W	1.53 μm	14.0 ppb @ 1 s 1.5 ppb @ 40 s	8.2 × 10^−9^
C_2_H_2_^71^	10.0 W	1.53 μm	7.0 ppb @ 1 s	4.4 × 10^−8^

As a typical example, the output power of the diode laser was amplified from 12.87 mW to 10 W, nearly an 800-fold increase by using an EDFA^71^. With the integration time of 1 s, the MDL of 7 ppb and normalized noise equivalent absorption (NNEA) coefficient of 4.4 × 10^−8^ cm^−1^ W/√Hz are achieved, demonstrating excellent detection performance for the C_2_H_2_-QEPAS sensor. These ultra-high sensitivity gas detection technologies confirm that amplifying the power of the excitation source by EDFA is one of the significant methods to improve QEPAS sensor performance.

#### Quantum cascade laser (QCL)-based QEPAS sensor

**Fig. 6:**
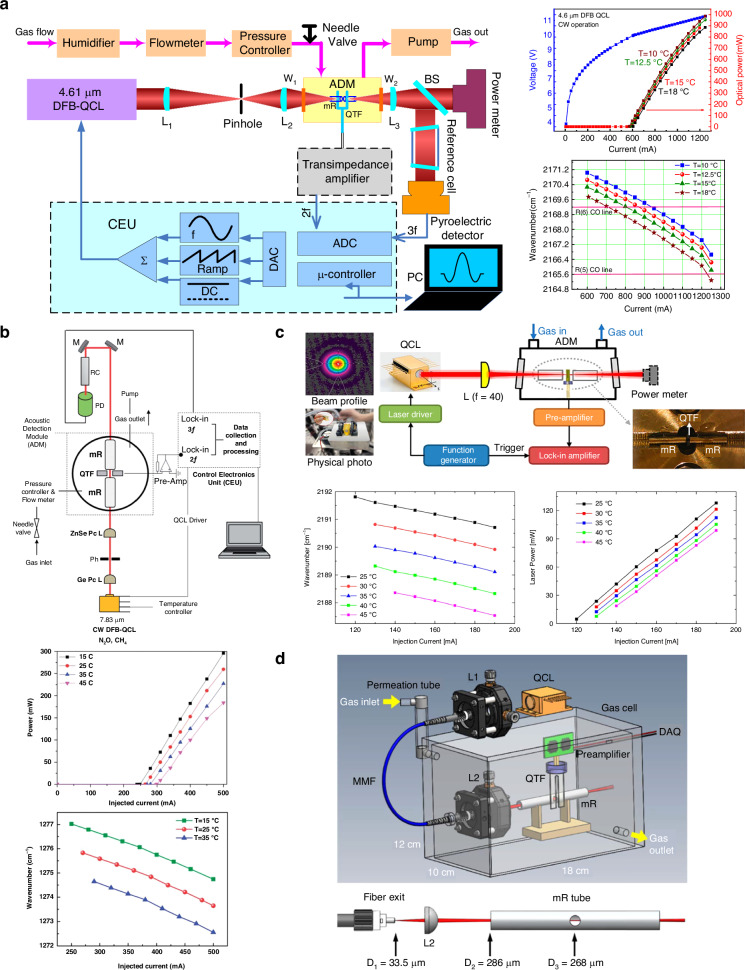
**Schematic of various types of QCL-based QEPAS sensors**. **a** CW-DFB-QCL with 1000 mW output power for CO detection^72^. **b** QCL with high heat load (HHL) package for CH_4_ and N_2_O detection^73^. **c** CW-DFB-QCL with butterfly package for N_2_O detection^76^. **d** Multimode fiber-coupled QCL for NO detection^81^. **a** is reprinted from ref. ^72^ with permission from the Optica Publishing Group. **b** is reproduced with permission from ref. ^73^ Copyright Royal Society of Chemistry. **c** is reprinted with permission from ref. ^76^. **d** is reprinted from ref. ^81^ with permission from the Optica Publishing Group

The QCL, a specialized diode laser that emits at mid-infrared and far-infrared bands, was first reported by Bell Laboratories in 1994. Due to their significant advantages of high optical power, wide tuning range, and mid-infrared output, QCLs have found widespread application in QEPAS sensors for detecting gases like carbon monoxide (CO), nitrogen dioxide (N_2_O), Freon 125, sulfur hexafluoride (SF_6_), H_2_S, and NH_3_ detection^72–80^. With output powers ranging from tens to hundreds of milliwatts, QCL-based QEPAS sensors can achieve MDLs in the range of tens of ppt to hundreds of ppb. The schematic configuration of various QCL-based QEPAS sensors is shown in Fig. 6. The parameters and performance of these sensors are summarized in Table 2.

**Table 2:** A summary of high-power QCL-based QEPAS sensors

Laser type	Target gas	Output power	Output wavelength	MDL @ Integration time	NNEA (cm^−1^ × W × Hz^−1/2^)
CW-DFB-QCL without package^72^	CO N_2_O	1000 mW	4.61 μm	340 ppt @ 500 s 4 ppb @ 500 s	1.6 × 10^−8^
CW-DFB-QCL with HHL package^73^	N_2_O CH_4_	>120 mW	7.83 μm	6 ppb @ 1 s 13 ppb @ 1 s	/
CW-DFB-QCL with HHL package^75^	SO_2_	155 mW	7.24 μm	63 ppb @ 1 s	1.2 × 10^−8^
CW-DFB-QCL with butterfly package^76^	N_2_O	>100 mW	4.56 μm	240 ppt @ 210 s	1.5 × 10^−8^
External cavity-QCL^80^	H_2_S	118 mW	7.90 μm	492 ppb @ 1 s	3.1 × 10^−9^
Multimode fiber-coupled based QCL^81^	NO	65 mW	5.26 μm	24 ppt @ 130 s	/

The distributed feedback QCL (DFB-QCL) is named for the distributed feedback grating embedded in the quantum cascade chip. It emits a narrow-linewidth, high-power single-mode laser with a high-purity spectrum. Wavelength tuning is achieved through temperature and current adjustments. In 2013, Ma used a continuous wave (CW)-DFB-QCL with an emission wavelength of 4.61 μm as the excitation source for measuring CO and N₂O. The laser has not been packaged, and its output power is close to 1000 mW. The experimental setup is shown in Fig. 6a. To enhance the vibrational-translational relaxation processes of CO and N₂O, water vapor was added within the QEPAS system. Finally, with an optimal integration time of 500 s, MDL of 340 ppt for CO and 4 ppb for N₂O were achieved^72^.

A methane (CH_4_) and N_2_O gas sensor based on QEPAS, utilizing a HHL-packaged CW-DFB-QCL operating at 7.83 μm, was proposed by Jahjah in 2014, as shown in Fig. 6b. The output power of the DFB-QCL exceeded 120 mW. A spatial filter, consisting of plano-convex lenses and a 200 μm pinhole, was employed to improve beam quality and focus the laser beam into the gas cell. The sensor achieved MDLs of 13 ppb and 6 ppb for CH_4_ and N_2_O detection, respectively, with a data integration time of 1 s^73^. In 2024, Ren et al. reported a N₂O-QEPAS sensor using a butterfly-packaged QCL emitting at 4.56 μm, as illustrated in Fig. 6c. This butterfly-packaged QCL offers a compact design with a small volume (4.95 cm^3^), lightweight (21 g), low threshold current (<120 mA), and high output power (>100 mW), making it well-suited for a compact QEPAS sensor^76^. Considering the optical fiber transmission and coupling not only boost the versatility of the QEPAS sensor but also achieve simple optical alignment, as shown in Fig. 6d, a multimode fiber-coupled-based QCL sensor for NO detection was reported by Ren et al. in 2016^81^. Applying an aspheric lens, the laser beam emitted from QCL was coupled into a solid-core InF_3_ multimode fiber with a core diameter of 100 μm, in which the coupling efficiency of 97%. Such a high delivery contributed an MDL of 24 ppb at an averaging time of 130 s.

#### Intracavity QEPAS sensor

**Fig. 7:**
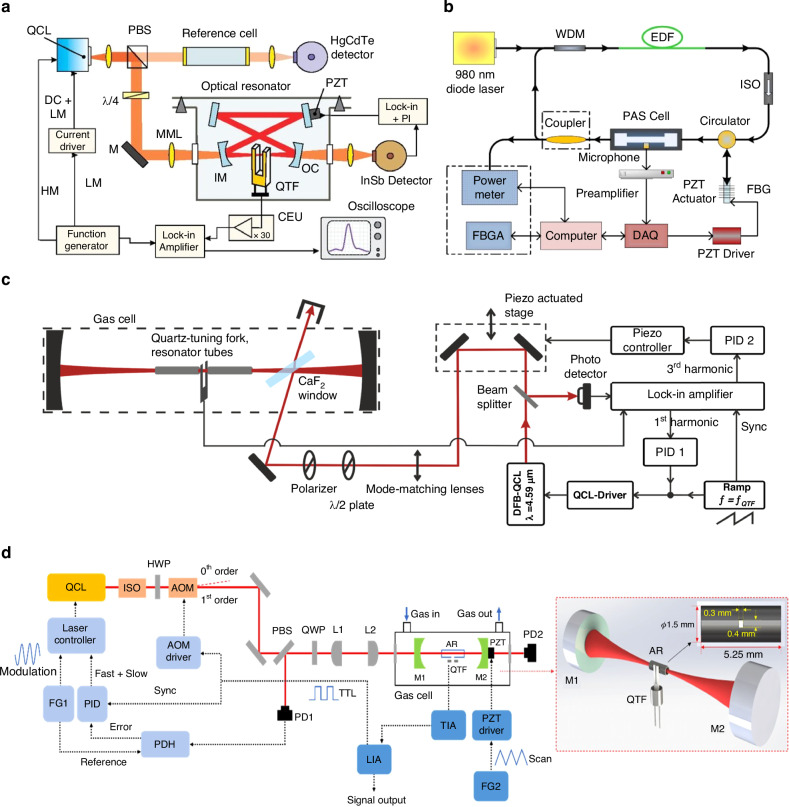
**Schematic of I-QEPAS sensors**. **a** The first proposed I-QEPAS sensor^82^. **b** The fiber ring laser-based I-QEPAS sensor^83^. **c** The I-QEPAS sensor for multicomponent detection^84^. **d** F-P cavity-based I-QEPAS sensor^85^. **a** is reproduced from ref. ^82^ with the permission of AIP Publishing. **b** is reprinted from ref. ^83^ with permission from the Optica Publishing Group. **c** is reproduced from ref. ^84^ with permission from Elsevier. **d** is reproduced from ref. ^85^ with the permission of AIP Publishing

Currently, the external-cavity configuration is commonly used in QEPAS sensors, where the QTF is positioned outside the laser cavity. In this external-cavity setup, the laser beam passes through the QTF gap only once, meaning the laser energy is utilized just once to excite the target gas and generate a photoacoustic signal. This leads to a low energy utilization rate and a weak signal, as the amplitude of the QEPAS signal is directly proportional to the laser excitation power. Additionally, this configuration has limited integration, which restricts the application of QEPAS sensors. To address these issues, intracavity QEPAS (I-QEPAS) was introduced by Borri et al.^82^. This technique combines the principles of cavity-enhanced absorption spectroscopy and QEPAS, leveraging the advantages of both methods. The schematic of the I-QEPAS sensor is shown in Fig. 7a. In this setup, an optical resonator with a bowtie shape is specifically designed for coupling with the QEPAS system. A QCL with a wavelength of 4.33 μm and an output power of 3 mW serves as the excitation source, matching the cavity mode. This results in an optical power enhancement by a factor of 250 (corresponding to a laser power of 750 mW) compared to the traditional external-cavity configuration. As a result, with an integration time of 20 s, the MDL for CO_2_ detection was achieved at 300 ppt, with a corresponding NNEA of 3.2 × 10^−10^ cm^−1^ W Hz^−1/2^.

Furthermore, it is also beneficial to realize tight integration. Several kinds of I-QEPAS constructions were presented, such as fiber ring laser intracavity and Fabry-Perot (F-P) intracavity. The fiber ring laser I-QEPAS sensor is shown in Fig. 7b, which was reported by Wang et al.^83^. Single-mode erbium-doped fiber was employed as the gain medium pumped by a 980 nm laser with a maximum power of 710 mW. A circulator was used to guarantee the circular transmission of the laser inside the fiber ring, which achieved a high-power density within the laser intracavity. A QEPAS detection module is placed inside the circulating cavity of the fiber ring laser. Therefore, the target gas can absorb a lot of laser energy and produce a strong photoacoustic signal. When the integration time of the system was set as 300 s, an MDL of 29 ppb was obtained for the detection of C_2_H_2_.

In 2022, Hayden reported a mid-infrared I-QEPAS sensor for multicomponent detection, with the schematic shown in Fig. 7b^84^. A DFB-QCL operating at a wavelength of 4.59 μm was selected as the excitation source, with CO, N₂O, and H₂O chosen as the target gases. The laser beam emitted by the DFB-QCL was coupled into the cavity through reflection from a CaF₂ window positioned at a Brewster angle, maximizing the power coupling. A high-finesse resonant cavity was employed to enhance the excitation power by a factor of 100, significantly improving the detection performance of the QEPAS sensor. The laser power inside the cavity was measured to be 25 mW for CO, 19.5 mW for N_2_O, and 19 mW for H_2_O detection. With an integration time of 10 s, the MDLs were achieved at 260 ppt for CO, 750 ppt for N_2_O, and 5.9 ppm for H_2_O, respectively. In a similar study on mid-infrared I-QEPAS sensors for CO detection, Ren et al. reported their findings in 2023, with the schematic depicted in Fig. 7b^85^. In this setup, an optical F-P cavity with a finesse of 1931 was used to enhance the laser power. To ensure stable detection across a broad spectral range, a dual-feedback Pound-Drever-Hall technique was employed to lock the frequency of the QCL tightly to the F-P cavity. With an incident laser power of 7.3 mW, the intracavity power exceeded 3 W, resulting in an amplification factor of more than 400. The achieved MDL was 375 ppb with an integration time of 150 s.

Regarding high-power excitation sources, pursuing higher power is the core trend for enhancing QEPAS performance. Directly using high-power mid-infrared light sources (such as QCLs), due to their dual advantages of combining high-power output with matching the strong absorption bands of gases, exhibits the most significant potential for performance improvement. This is currently the mainstream direction for the development of high-performance QEPAS systems. Optical amplifiers (such as EDFAs) are an effective supplementary means to increase the power of existing near-infrared systems, providing a relatively convenient upgrade approach. Although intracavity structures theoretically offer immense gain, their engineering complexity restricts their widespread application. In the future, as the cost of mid-infrared devices decreases and the technology matures, solutions based on high-power light sources like QCLs are expected to achieve broader application in terms of both sensitivity and practicality.

### Novel excitation source-based QEPAS sensor

Single-mode, narrow-bandwidth diode lasers are widely used in QEPAS sensors. However, due to their near-infrared output, these lasers can only target weak absorption lines in the overtone bands of gas molecules. The absorption cross-sections of these lines are 1–3 orders of magnitude weaker than those in the fundamental bands, which limits the detection performance of QEPAS sensors. With advancements in laser technology, various novel laser types, such as solid-state lasers, optical frequency combs (OFCs), and terahertz lasers, have been incorporated into QEPAS systems^86–93^. These innovations enhance the absorption strength, enabling highly sensitive gas detection.

#### Solid-state laser-based QEPAS sensor

**Fig. 8:**
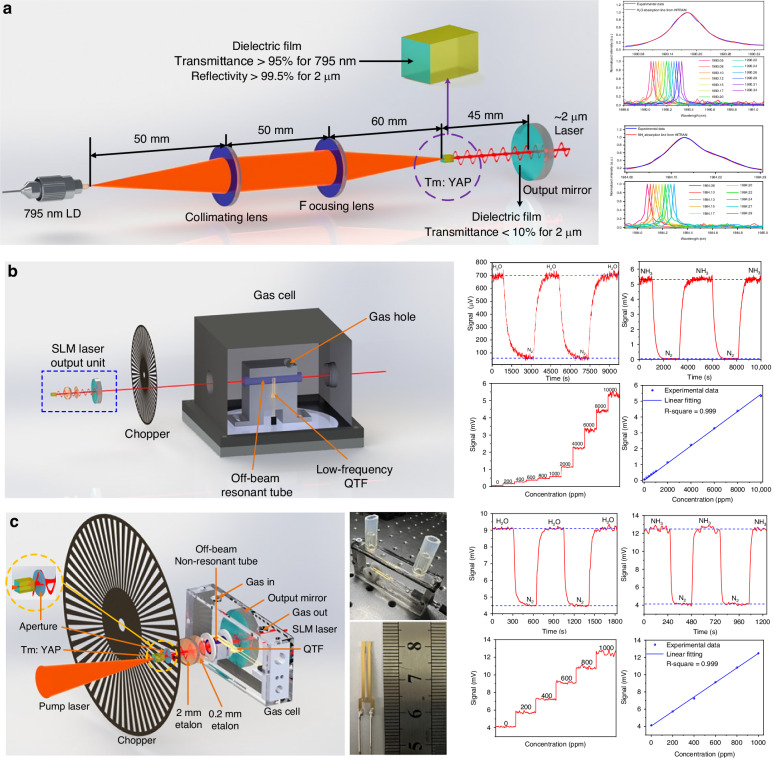
**Schematic of solid-state laser-based QEPAS sensor**. **a** The solid-state laser and its output characteristics^86^. **b** Solid-state laser-based external-cavity QEPAS sensor^86^. **c** Solid-state laser-based intracavity QEPAS sensor^86^. **a**–**c** are reprinted from ref. ^86^ with permission from Springer Nature by CC BY 4.0

Due to the excellent mode selection characteristics of solid-state lasers, they offer superior beam quality compared to other types of lasers. Additionally, by changing the laser gain medium, they can output long-wavelength laser emissions and achieve a wide tuning range. An attempt to employ a solid-state laser as the excitation source for a QEPAS system for NH_3_ and H_2_O detection was first demonstrated by Qiao in 2024^86^, with the schematic shown in Fig. 8a. The long-wavelength, single longitudinal-mode solid-state laser emitted at around 2 μm, with a maximum output power of approximately 130 mW and a wide tuning range of about 10 nm. The Tm:YAP crystal was selected as the gain medium, and a fiber-coupled diode laser with a center wavelength of 795 nm served as the pump source. To ensure high spectral purity, a double-etalon configuration was used. The linewidth of the laser output was approximately 1.85 pm, which is smaller than the absorption linewidth of the gas. Based on this solid-state laser excitation source, three QEPAS sensors were developed.

First, an external-cavity QEPAS sensor was investigated, as shown in Fig. 8b. An off-beam configuration was used to minimize thermal noise caused by laser irradiation and enhance the strength of the acoustic wave. The MDLs achieved were 57.3 ppm for H_2_O and 11.2 ppm for NH_3_. To increase the optical power, the gas cell was placed inside a resonant cavity, forming an I-QEPAS configuration, as depicted in Fig. 8c. A compact gas cell with dimensions of 70 × 14 × 31 mm was used. Due to the high optical power in the intracavity, the solid-state laser-based I-QEPAS sensor exhibited a 5-fold improvement in detection performance compared to the external-cavity configuration. Finally, for NH_3_ and H_2_O detection, the MDLs were improved to 2 ppm and 41.0 ppm, respectively.

#### Optical frequency comb-based QEPAS sensor

In recent years, OFC spectroscopy has emerged as a powerful tool across various fields, including gas sensing, spectral lidar, and hyperspectral holography. With its series of equally spaced, highly coherent frequency lines, OFCs provide extremely high-resolution molecular spectral information, offering a novel approach to high-precision spectral measurements^87^. Dual-comb spectroscopy (DCS) utilizes two optical comb sources with small repetition frequency differences, enabling asynchronous optical sampling and extracting spectral information from target molecules via multiple heterodyne interferences. This technique boasts advantages such as high resolution, broad spectral range, and rapid measurement capabilities^88,89^. Consequently, the use of OFCs as excitation sources enhances the sensitivity and multicomponent detection capabilities of gas sensing in QEPAS^90,91^.

**Fig. 9:**
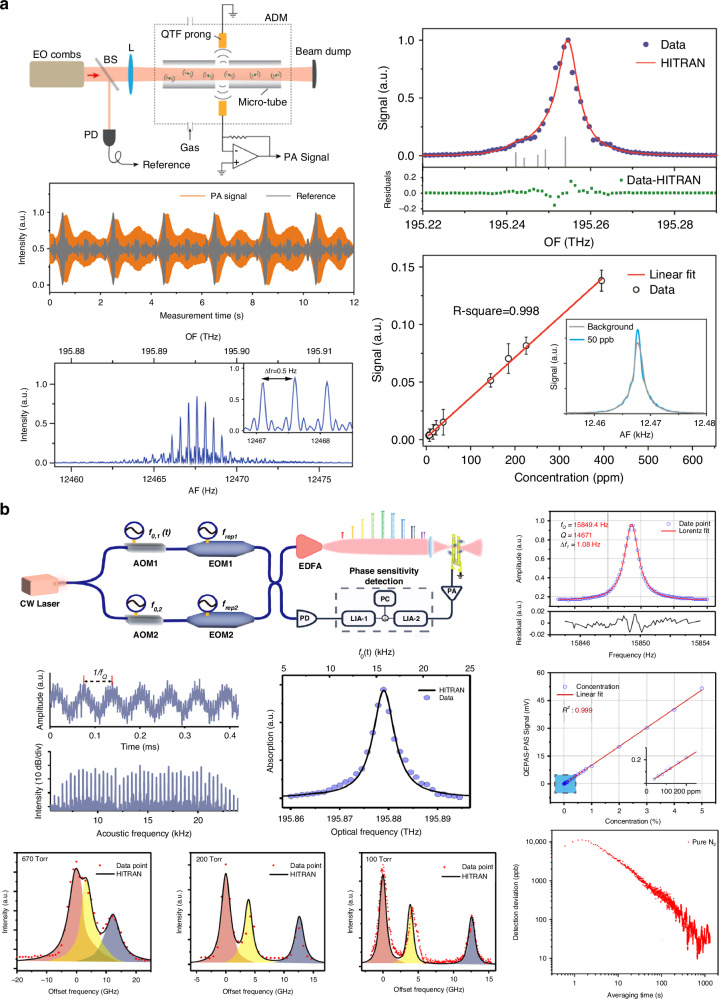
**Schematic of optical frequency comb-based QEPAS sensors**. **a** Dual-comb-based QEPAS^90^. **b** Dual-comb-based multi-heterodyne resonant QEPAS^91^. **a** is reproduced from ref. ^90^ with permission from Elsevier. **b** is reprinted from ref. ^91^ with permission from Springer Nature by CC BY 4.0

In 2022, Ren et al. reported a dual-comb-based QEPAS system, the schematic of which is shown in Fig. 9a^90^. The frequencies of the dual combs were tuned to 1530.37 nm for C_2_H_2_ detection. The dual electro-optic combs, with a relative coherence time exceeding 10 s, were split into two beams. One beam was focused by a lens into an ADM, which consisted of a QTF and a pair of acoustic micro-resonant tubes. The other beam was directed to a photodetector for reference. The dual combs covered a wide tunable wavelength range from 1520 nm to 1610 nm, and the comb line spacing ranged from 9 kHz to 1 GHz, facilitating high-resolution measurements. Additionally, the dual combs were amplified using an EDFA before entering the ADM, enhancing gas absorption and improving detection sensitivity. The detected signal was Fourier transformed to generate the radio frequency (AF) spectrum. Time-domain signals and frequency-calibrated spectra were acquired for a 2% C_2_H_2_ concentration. The noise equivalent concentration (NEC) was calculated to be 8.3 ppb, and the NNEA was 7 × 10^−10 ^cm^−1^ W Hz^−1/2^. Lastly, a mid-infrared OFC with a center wavelength of 3.3 μm was generated using difference frequency generation. CH_4_ was chosen as the target gas, and an NEC and NNEA of 0.5 ppm and 2.6 × 10^−10 ^cm^−1^ W Hz^−1/2^ were obtained, respectively.

Due to the narrow bandwidth of QTF, the number of available combs is limited. In addition, the requirement for coherence time is higher owing to the higher resolution caused by dense comb teeth. To address these challenges, Dong et al. proposed a quartz-enhanced multi-heterodyne resonant photoacoustic spectroscopy (QEMR-PAS) system in 2024, as shown in Fig. 9b^91^. Unlike traditional DCS, this approach recovers the resonant frequency components through multi-heterodyne processes. As a result, the time window limitation imposed by the Fourier transform method was eliminated, and the mutual coherence requirement between the dual combs was relaxed. The OFC generated using an electro-optic modulator consisted of 32 comb lines, each spaced 1 GHz apart. With the application of heterodyne QEPAS (H-QEPAS) technology, multi-heterodyne signals were generated. A custom QTF with a resonant frequency of 15.85 kHz was employed to detect the generated acoustic wave. The MDL for C_2_H_2_ detection was determined to be 50 ppb, with a linear dynamic range exceeding 40 dB. The spectral resolution of QEMR-PAS was evaluated by measuring the overlapping NH_3_ absorption spectrum, which contained three distinct absorption lines. Thanks to the narrow bandwidth of the QTF, the system successfully resolved the individual NH_3_ absorption lines.

#### Terahertz laser-based QEPAS sensor

Recently, advances in photonics and nanotechnology have driven the development of terahertz sources. Terahertz light has a longer wavelength than mid-infrared laser sources. In a QEPAS sensor, detection sensitivity is closely related to absorption strength, and many gas molecules exhibit strong absorption in the terahertz range. Therefore, using a terahertz excitation source offers significant advantages for enhancing the performance of QEPAS sensors^92,93^.

**Fig. 10:**
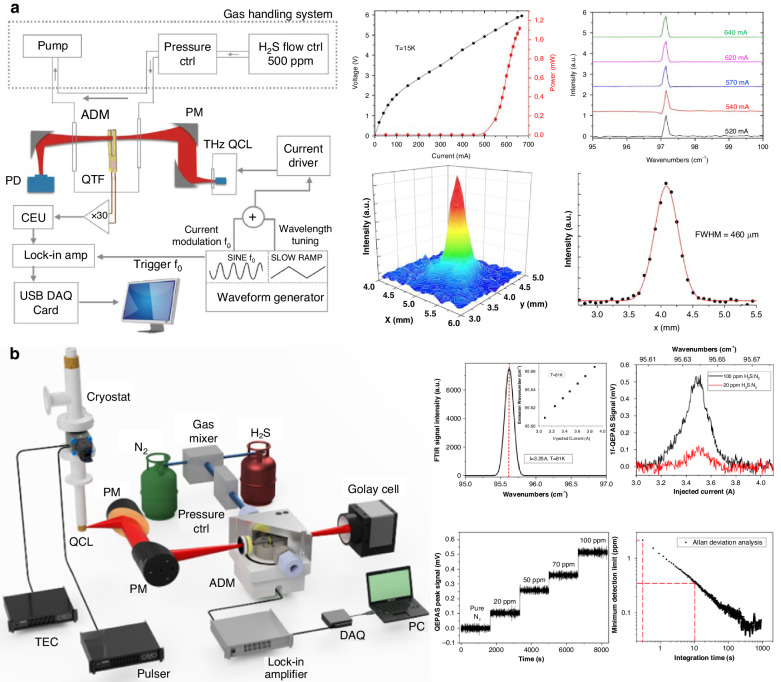
**Schematics of Terahertz laser-based QEPAS sensors**. **a** 102.98 μm THz laser-based QEPAS sensor for H_2_S detection^92^. **b** 104.57 μm THz QCL-based QEPAS sensor for H_2_S detection^93^. **a** is reprinted from ref. ^92^ with permission from the Optica Publishing Group. **b** is reproduced from ref. ^93^ with permission from Elsevier

In 2015, Vincenzo et al. proposed the first terahertz laser-based QEPAS sensor for hydrogen sulfide (H_2_S) detection, as illustrated in Fig. 10a^92^. A CW terahertz QCL operating at 2.913 THz (97.11 cm^−1^) with a power of 1.1 mW was used as the excitation source. Two 90° off-axis parabolic mirrors were employed to focus the terahertz laser beam onto the gap between the prongs of the QTF, where the power was measured to be 0.24 mW. It was confirmed that under optimal alignment conditions, 96.4% of the incident laser power was transmitted through the prong gap of the QTF. The obtained MDL was 13 ppm. In 2022, Sampaolo reported a terahertz QCL-based QEPAS sensor, as shown in Fig. 10b^93^. An absorption line of H_2_S at 95.626 cm^−1^ was selected. The system used an F-P THz QCL with a peak power of 150 mW in pulsed mode and an output wavelength of 104.6 μm. The ADM consisted of a low-frequency QTF in an off-beam mR-enhanced configuration. Intensity modulation was employed, and the 1*f*-QEPAS signal was demodulated. With an integration time of 10 s, an MDL of 360 ppb was achieved, corresponding to an NNEA of 3 × 10^−8^ cm^−1^ W Hz^−1/2^. The parameters and performance of QEPAS sensors based on these novel excitation sources are summarized in Table 3.

**Table 3:** A summary of novel excitation source-based QEPAS sensors

Method	Target gas	Output wavelength	MDL (@ Integration time)	NNEA (cm^–1^×W×Hz^–1/2^)
Solid-state laser-based QEPAS^86^	NH_3_	2.00 μm	NH_3_: 2.0 ppm @ 3 s H_2_O: 41.0 ppm @ 3 s	—
Optical frequency comb-based QEPAS^90^	C_2_H_2_ CH_4_	1530.37 nm 3.30 μm	8.3 ppb 0.5 ppm	7.00 × 10^−10^ 2.60 × 10^−10^
Optical frequency comb-based QEPAS^91^	C_2_H_2_	1531.46 nm–1539.54 nm	4.0 ppm @ 9.6 s 50.0 ppb @ 316 s	5.99 × 10^−8^
Terahertz laser-based QEPAS^92^	H_2_S	102.98 μm	30.0 ppm @ 3 s 13.0 ppm @ 30 s	4.40 × 10^−10^
Terahertz laser-based QEPAS^93^	H_2_S	104.57 μm	360.0 ppb @ 10 s	3.00 × 10^−8^

In the research of novel light sources, the core trend for performance enhancement is expanding the laser emission wavelength from near-infrared overtone absorption bands of gases to fundamental strong intrinsic absorption bands. This enables the generation of stronger detection signals under equal or superior optical power conditions, achieving a leap in sensitivity. Among the three summarized laser categories: (1) solid-state lasers currently represent the most practical solution for improving gas detection sensitivity. (2) OFCs demonstrate revolutionary potential in multicomponent, high-precision detection. (3) terahertz lasers provide a unique detection window for specific molecules. In terms of excitation source, future development directions will focus on optimizing the performance of these novel light sources, reducing costs, and resolving engineering integration issues to fully unlock their ultra-high sensitivity potential enabled by leveraging strong fundamental absorption.

### Custom QTF-based QEPAS sensor

For QEPAS sensors, the QTF is a critical component, and its performance significantly influences the detection sensitivity. One of the key parameters of a QTF is its resonant frequency (*f*_0_). In QEPAS systems, to achieve the strongest detection signal, the modulation frequency must match the *f*_0_ of the QTF, causing it to resonate and reach its maximum vibrational state. The *f*_0_ of the QTF determines the modulation period of the system, which in turn corresponds to the energy accumulation time for the target gases. Therefore, QTFs with a lower *f*_0_ allow for a longer interaction time between the laser and the gas, enabling the gas to absorb more laser energy and generate a stronger acoustic wave. As a result, the intensity of the detection signal is inversely proportional to the *f*_0_ of the QTF, as shown in Formula 1. Since the advent of QEPAS technology, standard commercial QTFs with a *f*_0_ of 32.768 kHz have commonly been used in sensor systems. However, such high frequencies reduce gas detection sensitivity. Additionally, some gases have slower relaxation rates. If the modulation period of the system is shorter than the lifetime of the upper energy level of these gases, weak or no photoacoustic signals are generated. Thus, QTFs with lower *f*_0_ values also offer advantages for detecting gases with slower relaxation rates. According to Euler-Bernoulli beam theory, the *f*_0_ of QTFs can be calculated using the following equation^94–96^:

2 $${f}_{0}=\frac{{n}^{2}\pi W}{8\sqrt{12}{L}^{2}}\sqrt{\frac{E}{\rho }}\propto \frac{W}{{L}^{2}}$$

where *n* is the number of vibration modes, *W* is the width of the prongs of QTFs, *L* is the length of the prongs of QTFs, *E* is Young’s modulus of materials, and *ρ* is the density of materials. To enhance detection performance, custom low-frequency QTFs have been designed and employed in QEPAS sensors since 2013^97^.

#### The design of QTF dimensions

**Fig. 11:**
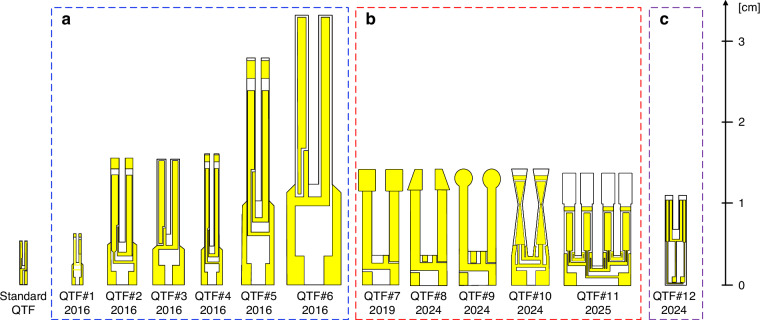
Schematics of custom QTFs. **a** Design of dimension. **b** Design of shape, **c** Design of material

**Fig. 12:**
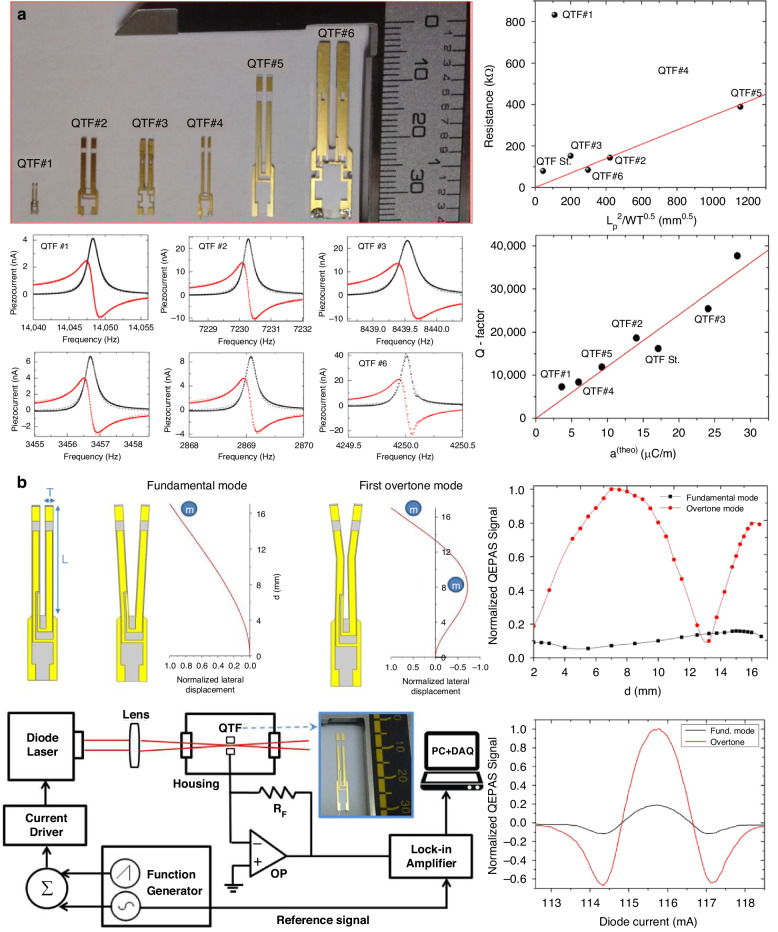
**Custom QTFs with different dimensions and the corresponding QEPAS sensing performance**. **a** Images of the actual QTFs and their related performance^98^. **b** QEPAS sensor exploiting overtone mode of custom QTF#5^94^. **a** is reproduced from ref. ^98^ with permission from Elsevier. **b** is reproduced from ref. ^94^ with permission of AIP Publishing

From Formula 2, it can be seen that low frequencies are primarily achieved by adjusting the width and length of the prongs of the QTFs. In 2016, V. Spagnolo et al. reported a series of custom QTFs with different geometric dimensions^98^. The schematic of these custom QTFs (QTF#1 to QTF#6) is shown in Fig. 11a, and their relevant parameters are listed in Table 4. The custom QTFs were fabricated from z-cut quartz wafers. Using photolithographic etching technology, chromium and gold patterns were deposited onto the sides of the quartz wafers. Experimental measurements of *f*_0_ were found to be in agreement with theoretical predictions, with only a 5% discrepancy. The results indicated that increasing the thickness (*T*) and width of the prongs, while reducing their length, improved parameters related to detection performance, including the Q-factor and electrical resistance. However, since *f*_0_ is proportional to *T*/*L*^2^, this led to an increase in *f*_0_, which is disadvantageous for performance improvement. These findings showed that the desired conditions could not be satisfied simultaneously, highlighting the need for a trade-off in the design of QTF dimensions to optimize detection performance. Figure 12a shows images of the actual QTFs (QTF#1 to QTF#6) and their related performance. To verify the improved sensing performance, a H_2_O-QEPAS sensor using custom QTF#5 was implemented, as shown in Fig. 12b^94^.

**Table 4:** The related parameters of custom QTFs

QTF type	Prong shape	Prong length (mm)	Prong width (mm)	Frequency (Hz)	Q-factor
Standard QTF	Rectangle	3.0	0.35	32,762.84	~12,000.00
QTF#1^98^	Rectangle	3.5	0.20	14,049.20	7323.49
QTF#2^98^	Rectangle	10.0	0.90	7230.27	18,654.18
QTF#3^98^	Rectangle	10.0	1.00	8439.51	25,484.95
QTF#4^98^	Rectangle	11.0	0.50	3456.69	8388.12
QTF#5^98^	Rectangle	17.0	1.00	2869.07	11,901.88
QTF#6^98^	Rectangle	19.0	1.40	4250.01	37,712.74
QTF#7^99^	T-shaped	9.4	2.00	12,460.55	15,540.00
QTF#8^100^	Trapezoidal-head	9.4	2.00	9471.67	11,411.00
QTF#9^100^	Round-head	9.1	2.20	9499.28	11,874.00
QTF#10^101^	Tapered	10.0	1.95	7830.64	6315.03
QTF#11^102^	Four-prong	9.4	1.60	7918.98	7763.00
QTF#12^103^	Lithium niobate	8.0	0.45	39,196.6	5900.00

The QTF was operated at both the fundamental and first overtone vibration modes, with *f*_0_ values of approximately 3 kHz and 18 kHz, respectively. Compared to the fundamental vibration mode, the first overtone mode exhibited a *Q*-factor approximately 2.6 times larger. The relationship between the QEPAS signal level and laser position along the prongs (from bottom to top) was investigated for both the fundamental and first overtone vibration modes. The optimal laser positions were found at distances of 15 mm and 7.5 mm for the fundamental and first overtone vibration modes, respectively. The QEPAS signal obtained in the first overtone vibration mode was 5.3 times stronger than that of the fundamental vibration mode.

#### The design of the QTF shape

**Fig. 13:**
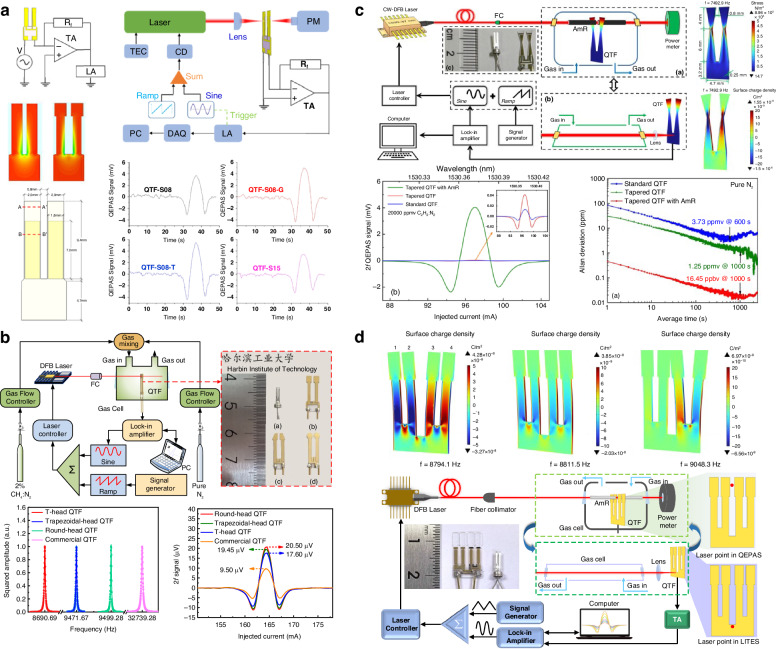
**The schematics of QEPAS sensors based on various shape custom QTFs**. **a** T-shaped custom QTF^99^. **b** Trapezoidal- and round-head QTFs^100^. **c** Tapered QTF^101^. **d** Four-prong QTF^102^. **a**, **b** are reprinted from ref. ^99^ and ref. ^100^, respectively, with permission from the Optica Publishing Group. **c** is reproduced from ref. ^101^ with permission of AIP Publishing. **d** is reproduced from ref. ^102^ with permission from the Opto-Electronic Journals Group

In addition to the *f*_0_, the piezoelectric conversion coefficient is another key parameter of the QTF that influences the detection performance of QEPAS sensors. Under the same excitation intensity, a QTF with a higher piezoelectric conversion coefficient can generate a stronger piezoelectric signal, resulting in better detection sensitivity. The most effective way to achieve this is by optimizing the geometric shape of the QTF prongs^99–102^. The goal of this approach is to maximize the internal stress generated in the QTF under the same excitation intensity, thereby producing a larger piezoelectric signal. QEPAS sensors based on custom QTFs with different shapes are illustrated in Fig. 13.

In 2019, a T-shaped QTF with low *f*_0_ was reported by Patimisco, and the related schematic is displayed in Fig. 13a^99^. The finite element software COMSOL Multiphysics was employed to complete the simulation of QTF, and the results indicated that compared with standard QTF, the T-shaped custom QTF produced a stronger stress field distribution along the surface of inner prongs. Moreover, to realize a custom QTF with a low electrical resistance, a groove-carved T-shaped QTF was designed. A H_2_O-QEPAS sensor was established to verify the detection performance of the designed QTFs. The experimental results indicated that compared to the QTF of the same size with right-angled prongs, the QEPAS system based on QTF with T-shaped prongs achieved a 1.5-fold enhancement of signal, due to a lower *f*_0_ and a stronger stress field distribution. Although the *f*_0_ and *Q*-factor were affected, the grooves-carved QTF-based QEPAS signal also realized a 1.36-times improvement, which demonstrated that the lower electrical resistance is more advantageous for detection performance.

In 2024, Ma et al. designed two novel QTFs with trapezoidal-head and round-head configurations, as shown in Fig. 13b^100^. According to simulation results, compared to the standard QTF, the average charge density and maximum stress distribution of the two novel QTFs improved by factors of 13.55 and 4.82, respectively. The performance of these two novel gold-coated QTFs was evaluated in a CH_4_-QEPAS sensor, showing SNR enhancements of 2.96-fold and 3.26-fold for the trapezoidal-head and round-head QTFs, respectively, compared to the standard QTF. Additionally, Ma et al. reported a tapered QTF with a low *f*_0_ of 7.83 kHz and a wide prong gap, as depicted in Fig. 13c^101^. This tapered QTF aimed to improve stress and charge distribution. In contrast to previous custom QTF designs, where prongs are typically rectangular and the maximum stress occurs at the root, the tapered design concentrated the maximum stress at the prong’s midpoint, enhancing both stress magnitude and charge distribution. Experimental tests demonstrated an SNR improvement of 3.02 times for the tapered QTF. Since the stress distribution of QTFs is focused on the internal side, where the support part connects prongs. The above-mentioned two-prong QTFs provide a narrow stress area. To enlarge the stress area and make the stress concentrated, a novel four-prong QTF with enlarged deformation area, large prong gap, and low resonant frequency was reported by Wang et al. and applied in the QEPAS system, as illustrated in Fig. 13d^102^. According to the simulation, in contrast with the standard two-prong QTF, the maximum stress and surface charge density had 11.1-fold and 15.9-fold enhancements. In experiments, the C_2_H_2_-QEPAS system contributed to verify the performance of the proposed four-prong QTF. Compared with the standard two-prong QTF, the four-prong QTF exhibited a 4.67 times enhanced signal-to-noise with the same conditions.

#### The design of the QTF material

**Fig. 14:**
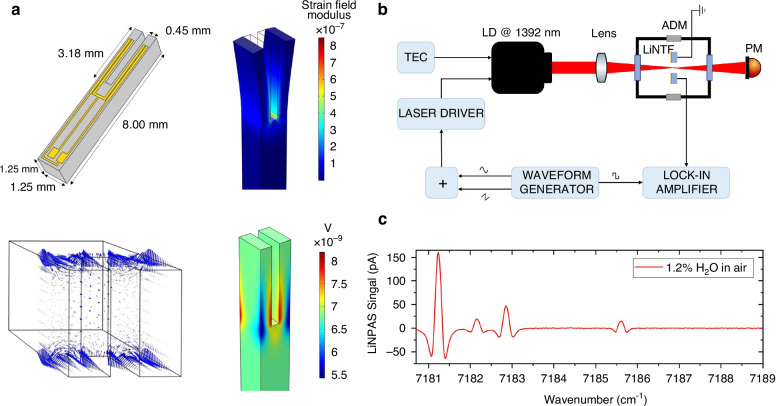
**Schematics of LiNTF-based QEPAS sensor**. **a** Simulation results^103^. **b** Experimental setup^103^. **c** The measured 2*f* signal with 1.2% H_2_O^103^. **a**–**c** are reproduced from ref. ^103^ with permission from Elsevier

Piezoelectric materials are typically limited to quartz for manufacturing custom QTFs. However, other materials, such as lithium niobate (LiN), have been widely used in various piezoelectric devices but are rarely employed in tuning forks (TFs). Compared to quartz, LiN exhibits a tenfold larger effective piezoelectric coefficient, suggesting that LiN-based tuning forks (LiNTFs) could deliver superior detection performance. In 2024, Cantatore et al. proposed a novel LiNTF, as shown in Fig. 14^103^. The geometry of the LiNTF is similar to that of a standard QTF, and it was fabricated using a 128° Y-cut LiN wafer. The strain field is distributed across the inner surfaces of the prongs, while the electric field is concentrated on the front surface of the LiNTF. The *f*_0_ and Q-factor were measured at 39.1966 kHz and 5900, respectively. To assess the detection performance, a QEPAS sensor was developed using the LiNTF-based sensing system, with water vapor chosen as the target. A diode laser with a center wavelength of 1392 nm and an output power of 10 mW was used as an excitation source. Allan deviation analysis revealed an MDL of 2 ppm at an integration time of 20 s.

In quartz-enhanced laser spectroscopy sensing technology, QTFs serve as the core detection element of the system, whose parameters play a decisive role in the system’s detection performance. Currently, QTF optimization primarily focuses on designing the QTF’s geometric dimensions, the shape of its prongs, and modifying the base material of the fork. Among these, designing the geometric dimensions of the fork aims to reduce its resonant frequency, thereby increasing the system’s energy accumulation time and enabling the detection of gas molecules with low relaxation rates. Designing the shape of the fork prongs and changing the base material aims to improve charge conversion efficiency, allowing for stronger detection signals under the same excitation. Future QTF optimization designs can further progress from macro-scale dimensional design towards micro/nano-scale precision manufacturing and structural innovation. Centered on maximizing detection sensitivity and minimizing system volume/complexity/cost, this will expand the capabilities of quartz-enhanced laser spectroscopy sensing technology for a wider range of gas species, more complex environments, and broader application scenarios.

### Acoustic wave-enhanced QEPAS sensor

In QEPAS technology, QTFs serve as acoustic transducers to detect the photoacoustic signals generated by target gases. A stronger acoustic signal can excite the QTF, causing greater vibrational displacement and producing a larger piezoelectric response. In addition to increasing the excitation and detection power, enhancing the intensity of the acoustic wave can also improve the signal level and overall detection performance. The enhancement of acoustic waves can be approached in two ways: first, by increasing the effective interaction time between the acoustic waves and the QTF prongs; second, by strengthening the acoustic wave intensity using acoustic resonator enhancement techniques to form a standing wave field^104–114^.

#### Multi-pass-enhanced QEPAS sensor

In traditional QEPAS sensors, the laser is directed perpendicular to the plane of the QTF, passing through the gap between its prongs only once. The QTF is excited by a single-point sound source with relatively low excitation intensity. One effective method to enhance the intensity of the acoustic signals is to design and optimize the optical path of the system. This approach aims to increase the interaction between the laser and the gases, thereby generating multiple or stronger acoustic excitations.

**Fig. 15:**
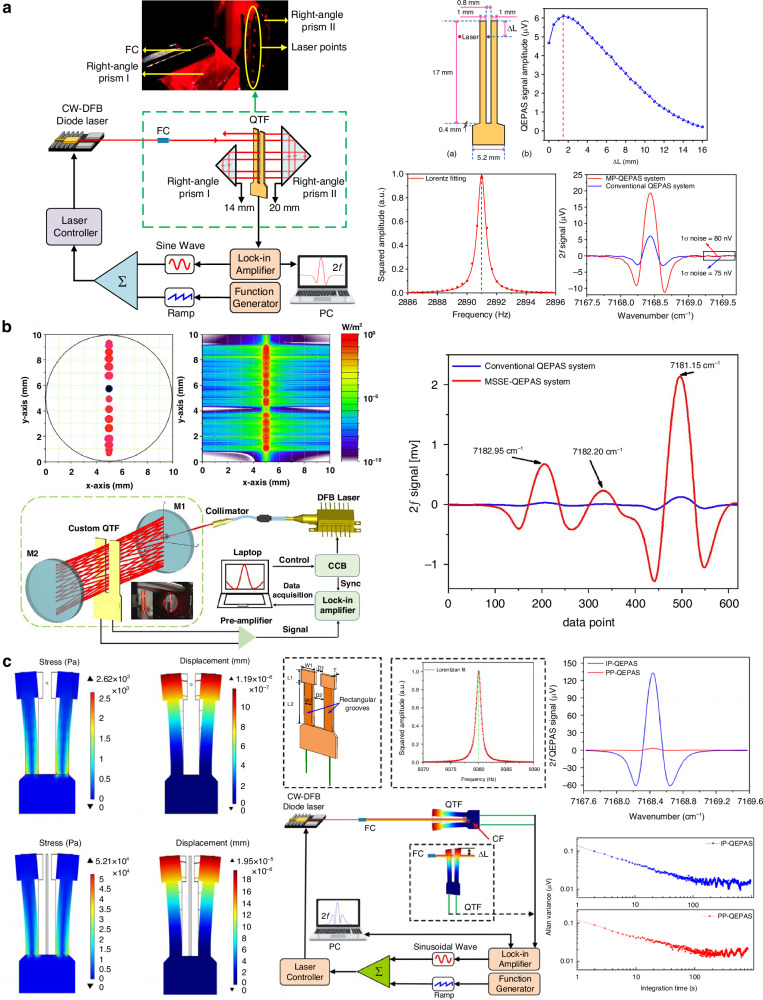
**The schematics of multi-pass-enhanced QEPAS sensors**. **a** Multi-pass QEPAS sensor^104^. **b** Multiple-sound-source-excitation QEPAS based on a single-line spot pattern multi-pass cell^105^. **c** In-plane QEPAS^106^. **a** is reprinted from ref. ^104^ with permission from the Optica Publishing Group. **b**, **c** are reproduced from ref. ^105^ and ref. ^106^, respectively, with the permission of AIP Publishing

In 2021, Qiao et al. reported a multi-pass configuration-based QEPAS sensor, as shown in Fig. 15a^104^. To ensure that the laser beam passed through the gap between the QTF prongs multiple times, two right-angle prisms with lengths of 14 mm and 22 mm were used. This multi-pass configuration allowed the laser to pass through the QTF prong gap six times, generating multiple acoustic wave sources that synergistically enhanced the excitation of the QTF. When water vapor was used as the target gas, the 2*f* signal of the multi-pass QEPAS sensor showed a 3.2-fold enhancement compared to the standard single-pass QEPAS sensor. An improved multiple-sound-source-excitation QEPAS (MSSE-QEPAS) sensor was proposed by Cui et al.^105^. It integrated a single-line spot pattern multi-pass cell (MPC), as shown in Fig. 15b^105^. The MPC consisted of two spherical mirrors with diameters of 10 mm and curvature radii of 100 mm. The laser beam transmitted through the MPC was aligned in the same plane, which facilitated its passage through the gap of the QTF prongs. This design enabled 60 passes of the laser beam through the QTF gap, generating multiple sound sources that enhanced the vibration of the QTF prongs, thereby boosting the QEPAS signal. Experimental results showed that this MSSE-QEPAS sensor achieved a 19-fold enhancement in signal level.

In 2020, Ma et al. reported the in-plane QEPAS (IP-QEPAS), as shown in the schematic in Fig. 15c^106^. In this approach, the laser beam was aligned to lie in the same plane as the QTF, and the line sound source was generated along the entire length of the prongs. Simulation results indicated that, compared to traditional QEPAS, the IP-QEPAS exhibited more than an order of magnitude improvement in both stress field and displacement. Experimental investigations of the IP-QEPAS sensor showed that, compared to the traditional QEPAS sensor, this approach achieved more than a 40-fold enhancement in signal level.

#### Acoustic resonator-enhanced QEPAS sensor

**Fig. 16:**
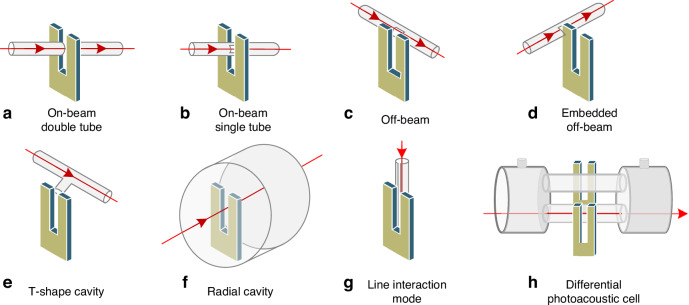
A summary of acoustic resonator enhancement methods for QEPAS sensor. **a** On-beam double tube mode. **b** On-beam single tube mode. **c** Off-beam mode. **d** Embedded off-beam mode. **e** T-shape cavity. **f** Radial cavity. **g** Line interaction mode. **h** Differential photoacoustic cell

Enhancing the amplitude of acoustic waves can significantly improve the detection performance of a QEPAS sensor. Similar to resonant photoacoustic cells in PAS, adopting a one-dimensional acoustic resonator to form a standing wave field for boosting acoustic wave amplitude is also effective in QEPAS. This approach is referred to as acoustic resonator-enhanced QEPAS. Various types of acoustic resonant cavities have been reported, including on-beam, off-beam, and T-shaped configurations, among others^107–114^. A summary of these acoustic resonator enhancement methods for QEPAS sensors is presented in Fig. 16.

**Fig. 17:**
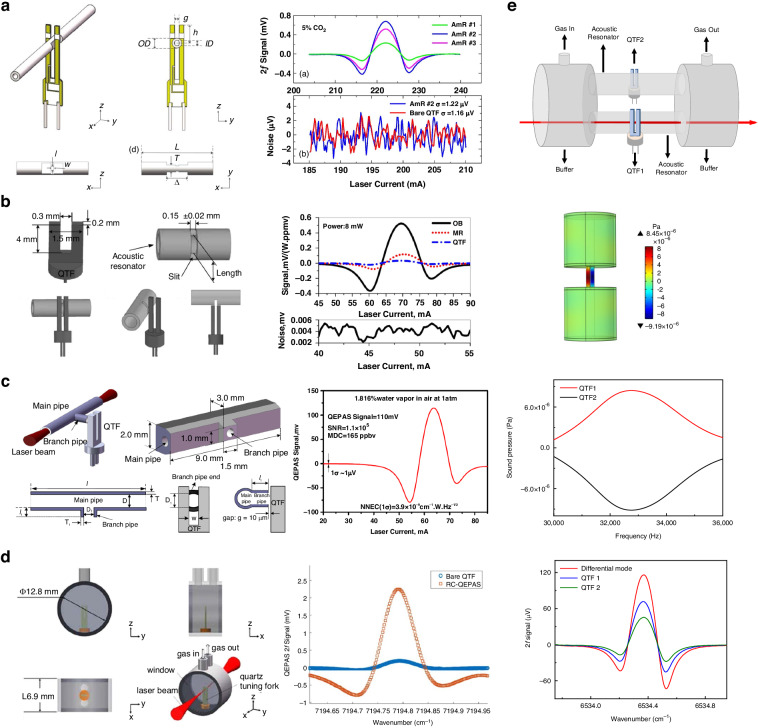
**The schematics of acoustic resonator-enhanced QEPAS sensors**. **a** Single-tube on-beam based QEPAS^108^. **b** Off-beam based QEPAS^109^. **c** T-shape AmR based QEPAS^111^. **d** Radial-cavity-based QEPAS^112^. **e** Differential photoacoustic cell-based QEPAS^114^. **a**–**d** are reprinted from refs. ^108,109,111,112^, respectively, with permission from the Optica Publishing Group. **e** is reproduced from ref. ^114^ with the permission of AIP Publishing

The most classic acoustic resonator-enhanced QEPAS configuration is the on-beam design, as illustrated in Fig. 16a. This configuration consists of two discrete resonant tubes positioned on either side of the QTF, forming an acoustic micro-resonator (AmR) that is perpendicular to the QTF plane. The laser beam passes through the gap between the QTF and the resonant tubes. According to acoustic resonance theory, the acoustic wave resonates within the tubes, creating a standing wave field. The lengths of the two resonant tubes should fall within the range of one-quarter (*λ*_s_/4) to one-half (*λ*_s_/2) of the wavelength of the generated acoustic wave. The on-beam configuration offers a maximum 30-fold signal enhancement^107^. However, due to the use of two separate resonant tubes, the acoustic coupling efficiency between the tubes and the QTF is relatively low. As custom QTFs with larger prong spacing were developed, it became possible to place a single resonant tube in the prong gap, eliminating the need for two separate tubes. In 2016, Zheng et al. proposed a single-tube on-beam QEPAS (SO-QEPAS), shown schematically in Fig. 17a^108^. The single-tube AmR offers advantages such as shorter length, which simplifies laser alignment and reduces background thermal noise. A custom QTF with a large prong spacing of approximately 800 μm was used in this SO-QEPAS sensor. The optimized AmR had an outer diameter of 0.9 mm, an inner diameter of 0.65 mm, and a length of 38 mm. This configuration resulted in a 135-fold signal enhancement compared to the bare QTF without the single-tube AmR.

However, the on-beam QEPAS requires precise alignment of the laser beam. To address this challenge, Liu et al. proposed an off-beam (OB-QEPAS) configuration featuring a unique AmR in 2009, as shown in Fig. 17b^109^. In the OB-QEPAS setup, the laser beam is directed along the resonant tube and does not pass through the gap between the QTF prongs. Based on the theory of the first longitudinal standing wave field, the length of the AmR is set to half the wavelength of the generated acoustic wave (*λ*_s_/2). A slit is incorporated in the center of the AmR to release sound waves, allowing the QTF to detect them. The signal enhancement achieved with this configuration was 15.7 times greater compared to a bare QTF in a standard QEPAS setup. The off-beam configuration introduces a simpler approach to laser alignment. However, its sensitivity is generally considered lower than that of the on-beam configuration.

Compared to the on-beam and off-beam configurations, the on-beam configuration is advantageous for enhancing detection capability, while the off-beam configuration simplifies laser alignment. Inspired by a T-shaped photoacoustic cell, Li et al. proposed a T-shaped AmR in 2012, which combines the benefits of both the on-beam and off-beam configurations^111^. The schematic of this setup is shown in Fig. 17c. In the T-shaped AmR, a branch pipe is inserted into and connected with the main pipe at the center. The laser beam passes through the main pipe, while the QTF is positioned at the side of the branch pipe. Experimental results show that this T-shaped AmR achieves a 30-fold signal enhancement compared to a bare QTF, similar to the on-beam configuration. At the same time, it simplified the laser alignment process, retaining the advantages of the off-beam configuration. Radial-cavity-based QEPAS (RC-QEPAS) was introduced by Zheng et al. in 2019, with its schematic diagram shown in Fig. 17d^112^. In RC-QEPAS, the resonance mode is radial. Compared to the longitudinal resonators discussed earlier, the radial resonator offers several advantages, including easier alignment, stronger resonance, and a more compact size. This RC-QEPAS configuration demonstrated a performance improvement of one order of magnitude over the bare QTF. Generally, due to the narrow inner diameter of most AmRs, thermal noise caused by light radiated onto the tube wall tends to increase. To mitigate this issue, one solution is to use a photoacoustic cell that achieves the same acoustic resonance effect as an AmR. In 2023, Ma et al. proposed a differential QEPAS, illustrated in Fig. 17e^114^. The differential photoacoustic cell consists of two identical resonant cavities with opposite phases and equal amplitudes, along with two buffer chambers. Two QTFs, with a *f*_0_ of 3 Hz, are placed at the center of the two resonant cavities to detect the photoacoustic signal. Compared to a single QTF, this configuration results in a 1.65-fold increase in signal amplitude.

In QEPAS systems, the detection performance is directly influenced by the intensity of the acoustic waves acting between the prongs of the QTF: The stronger the acoustic signal, the greater the excited vibrational displacement of the QTF, and the more pronounced the generated piezoelectric response, thereby enhancing the signal level and overall sensitivity of gas detection. Therefore, optimizing the interaction between the acoustic wave and the QTF is a key pathway to enhancing detection performance. The core strategy focuses on two points: firstly, enhancing the interaction between the laser and the gas to obtain a strong excitation acoustic source; and secondly, utilizing an acoustic resonator to form a high-intensity standing wave field to amplify the acoustic wave energy. Both methods can effectively increase the intensity of the acoustic waves acting on the QTF. In addition, these two methods can also be combined to jointly drive significant improvements in the system’s SNR and detection limit.

## LITES-based gas sensing

### The principles of LITES

**Fig. 18:**
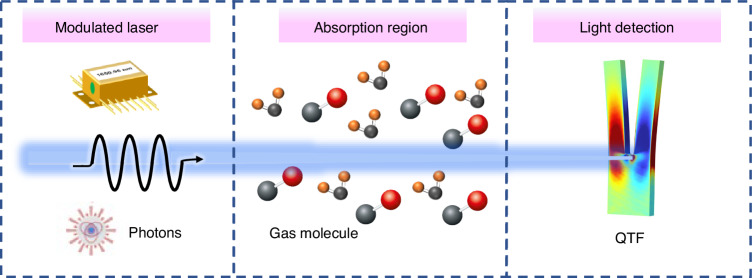
The principle of LITES technology

LITES technology not only retains the advantages of QEPAS but also enables non-contact gas measurement, which is vulnerable to damage from corrosive gases or harsh combustion environments. The key principle behind LITES technology is the use of the light-induced thermoelastic effect in the QTF to detect changes in laser intensity, as illustrated in Fig. 18. The modulated laser, after being absorbed by the gas, is focused onto the base of the QTF. The quartz substrate of the QTF absorbs the light energy, causing localized thermal-elastic expansion, which in turn induces periodic mechanical deformations. These deformations are then converted into an electrical signal through the piezoelectric effect of the QTF, allowing for gas concentration analysis^45^. Based on this principle, the sensor output of the LITES system can be expressed as:

3 $$S=kQ{I}_{2\omega }$$

where *k* represents the system constant, *Q* denotes the Q-factor of the QTF, and *I*_*2ω*_ refers to the optical power perturbation, which represents the periodic changes in laser power caused by gas absorption and laser modulation. This formula provides guidance for improving the detection performance of the LITES system.

Significant advancements have been made in recent years to enhance the performance of LITES sensors. The optimization of gas sensor sensitivity based on LITES has primarily focused on improving optical absorption, enhancing QTF detection performance, and increasing system response speed. From the structure of the LITES system, it can be seen that laser sources and QTFs are two core components. The parameters of both have a decisive influence on the detection performance. As a type of laser absorption spectroscopy technology, the core optimization strategy for the excitation sources in LITES, consistent with QEPAS technology, remains focused on enhancing the laser energy absorbed by the target gas. Specific methods include employing an EDFA-amplified laser and extending the detection wavelength using laser sources such as QCL. Regarding the detection element, custom QTFs are also an effective way to enhance the performance of the LITES system. For example, the resonant frequency can be lowered by adjusting the QTF dimensions, or the charge conversion efficiency can be improved by designing the QTF shape and base material. This aligns with the optimization approaches in QEPAS technology, and the custom QTFs shown in Fig. 11 are similarly applicable for enhancing the detection performance of the LITES system. Therefore, to avoid repetition with the discussion in the QEPAS technology section, this paper will focus on performance enhancement approaches beyond laser source optimization and QTF customization in the section on LITES technology.

**Fig. 19:**
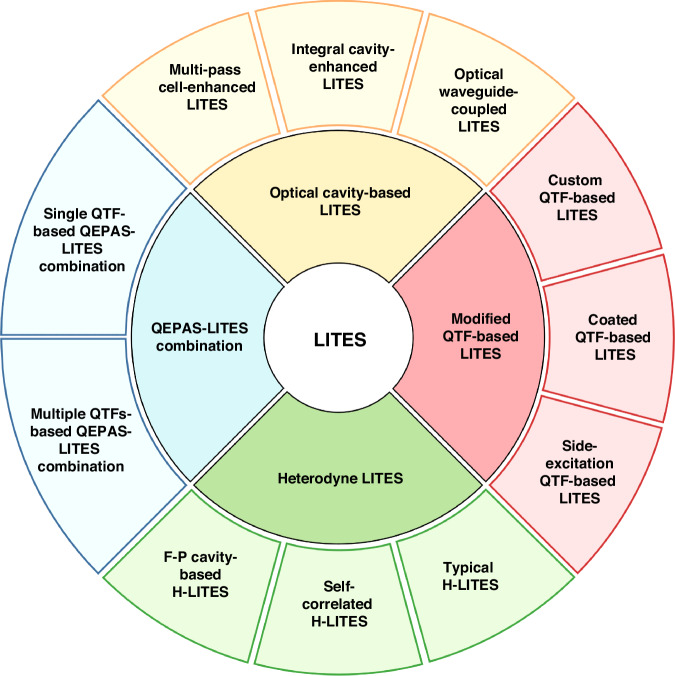
A summary of various approaches for improving LITES technology

Based on the above analysis, as shown in Fig. 19, the other effective strategies for optimizing the detection performance of LITES sensors can be categorized as follows: (1) employing cavity enhancement techniques to boost absorbance; (2) improving detection capabilities through QTF modification or the use of custom QTFs; (3) utilizing the transient response characteristics of QTFs to build a heterodyne system to enhance the response speed of the LITES sensor; and (4) leveraging the advantages of QEPAS technology to maximize laser energy utilization.

### Optical cavity-based LITES sensor

By employing various cavity designs, such as Herriot MPCs, dense-spotted MPCs, and off-axis integral cavities, the number of light beam reflections within the cavity can be increased, thereby enhancing the interaction between light and gas and significantly improving the detection performance of the LITES sensors. In addition to enhancing absorption signals with optical cavities, the use of optical waveguides for light transmission can substantially reduce sensor size and improve their integrability.

#### Multi-pass cell-enhanced LITES sensor

MPCs can significantly enhance light absorption by creating multiple reflections of the laser beam through high-reflectivity mirrors. In 2019, He et al. proposed an ultra-sensitive LITES sensor based on a Herriott MPC^45^.

**Fig. 20:**
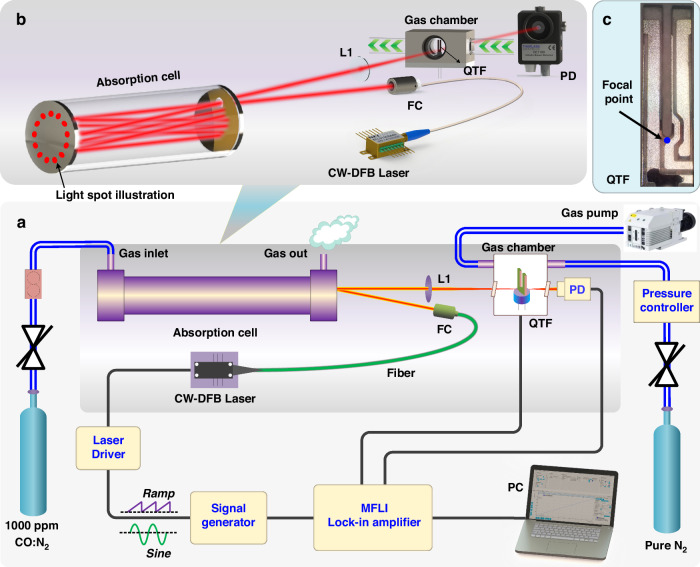
Herriott MPC-based LITES sensor. **a** Schematic of a LITES sensor system^45^. **b** The particular optical path structure^45^. **c** The image of the used QTF^45^. **a**–**c** are reprinted from ref. ^45^ with permission from the Optica Publishing Group

**Fig. 21:**
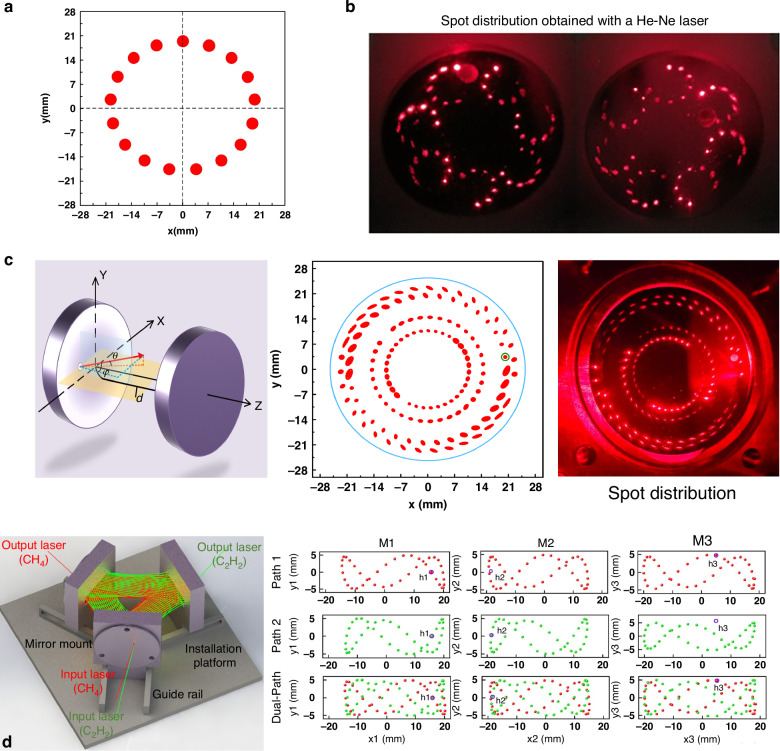
**Absorption-enhanced LITES sensors based on different types of MPCs**. **a** Spot distribution diagram of Herriott MPC. **b** Spot distribution images of MPC with a seven-nonintersecting-circle spot pattern^115^. **c** Structure parameter diagram, simulated and actual spot distribution images of MPC with four concentric rings^116^. **d** Structural schematic diagram and simulated spot distribution images of MPC with Lissajous spot pattern^117^. **b** is reproduced with permission from ref. ^115^ Copyright American Chemical Society. **c**, **d** are reproduced from ref. ^116^ and ref. ^117^, respectively, with permission from the Opto-Electronic Journals Group

As shown in Fig. 20, a laser beam with a central wavelength of 2330.19 nm passes through a gas absorption cell with an effective optical path length (OPL) of 10.1 m. The typical spot diagram is depicted in Fig. 20a. Experimental results demonstrated that an MDL of 17 ppb for CO detection could be achieved. The widely used Herriott MPCs involve the laser beam reflecting between two spherical mirrors, typically forming single circular or elliptical spot patterns, as shown in Fig. 21a. These spots occupy only a small fraction of the mirror surface area, leading to low mirror utilization. To address this issue, Cui et al. proposed a dense-spot MPC in 2020^115^, with simulated and actual spot patterns at the laser entry and exit ports shown in Fig. 21b. The optical path is optimized to generate multiple new spots on the mirror surface after each reflection, ensuring that these spots do not overlap and fully utilize the mirror area. This design enhances the efficiency of the laser beam’s interaction with the gas. In 2024, Liu et al. introduced an ultra-sensitive LITES sensor based on an MPC with a dense-spot pattern^116^, as shown in Fig. 21c. The OPL and the ratio of optical path length to volume (RLV) of the MPC are 37.7 m and 13.8 cm^−2^, respectively, representing approximately an eightfold improvement over traditional Herriott cells. The MDL for C_2_H_2_ detection is as low as 24.6 ppb.

Also in 2024, Sun et al. proposed an MPC based on Lissajous space-division multiplexing technology^117^, as shown in Fig. 21d. The reported MPC with Lissajous spot patterns is formed by using three reflective mirrors with different incident angles and mirror spacings. This configuration results in an OPL of 9.1 m and 9.0 m, and RLV values of 13.5 cm^−2^ and 13.3 cm^−2^, respectively. This design not only increases the OPL but also enhances the compactness of the system. After optimizing the system parameters, detection of CH_4_ and C_2_H_2_ achieved MDLs of 268.8 ppb and 91.4 ppb, respectively, demonstrating excellent sensitivity.

A comparison of absorption-enhanced LITES sensors based on MPCs is presented in Table 5. These studies, by utilizing MPCs with a dense-spot distribution pattern, not only optimize the laser beam’s distribution within the cavity but also significantly increase the effective OPL of the laser interacting with the gas. This, in turn, greatly enhances both the detection performance and the compactness of the LITES system.

**Table 5:** Comparison of MPC-enhanced LITES sensors

Optical cavity	*N*	OPL (m)	RLV (cm^−2^)	Target gas	Gas absorption line (cm^−1^)	MDL @ Integration time
Herriott MPC^45^	34	10.1		CO	4291.50 cm^−1^	470.0 ppb @60 ms
3D-printed MPC with dense spot^115^	107	4.2	21.0	CH_4_	6046.95 cm^−1^	52.0 ppb @300 ms
MPC with four concentric rings spot^116^	274	37.7	13.8	C_2_H_2_	6534.37 cm^–1^	24.6 ppb @240 ms
MPC with Lissajous pattern spot^117^	120	9.1	13.5	CH_4_	6057.08 cm^−1^	268.8 ppb
	119	9.0	13.3	C_2_H_2_	6534.37 cm^−1^	91.4 ppb

*N* reflection number, *OPL* optical path length, *RLV* ratio of optical path length to volume

#### Integral cavity-enhanced LITES sensor

**Fig. 22:**
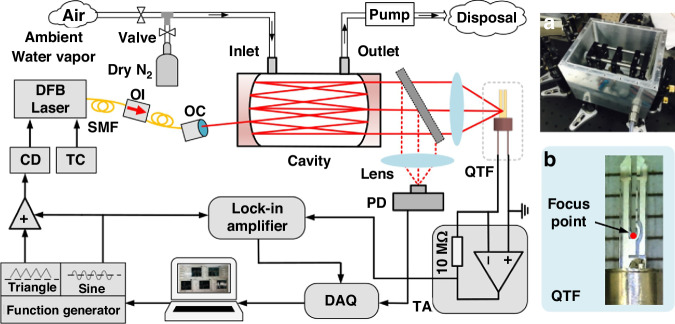
**Experimental configuration of the Off-axis integral cavity-enhanced LITES sensor**. **a** The fabricated cage-based cavity^118^. **b** The used QTF with an optimal focus point^118^. **a**, **b** are reprinted from ref. ^118^ with permission from the Optica Publishing Group

An off-axis integral cavity can also be employed to increase the OPL. Unlike the MPC configuration, the laser beam is directed into the interior of the off-axis cavity at a specific angle using high-reflectance mirrors. This setup creates a stable, non-axial propagation path within the cavity, thereby enhancing the effective OPL for interaction between the laser and the absorbing gas. In 2021, Zheng et al. demonstrated a highly sensitive LITES sensor utilizing an off-axis cavity^118^. As shown in Fig. 22a, the high-finesse off-axis integral cavity, with a cavity length of approximately 10 cm, achieves an effective OPL of 15 m, equivalent to about 150 times the intracavity laser propagation distance. This configuration not only improves the sensor’s sensitivity but also reduces the free spectral range and optical noise, thanks to its structural advantages. Consequently, it optimizes both the cavity mode structure and the mechanical stability of the system. Experimental results confirmed the significant enhancement in detection performance due to the off-axis cavity design in the LITES system.

#### Optical waveguide-based LITES sensor

Although MPCs have been instrumental in enhancing sensitivity, their size and the complexity of optical alignment have limited their potential for integration and miniaturization. In contrast, the use of optical waveguides as gas cells, thanks to their compact structure, simplified optical alignment, and low transmission loss, provides a promising pathway for miniaturizing and integrating LITES sensors. In 2022, Ma et al. reported a LITES sensor based on a hollow-core anti-resonant fiber (HC-ARF) gas sensor^119^. Hollow-core fibers, as an innovative optical waveguide technology, enable efficient long-distance transmission of light signals. The reported sensor used a customized silica-based HC-ARF, 75 cm in length, which served both as the optical medium and the gas cell.

**Fig. 23:**
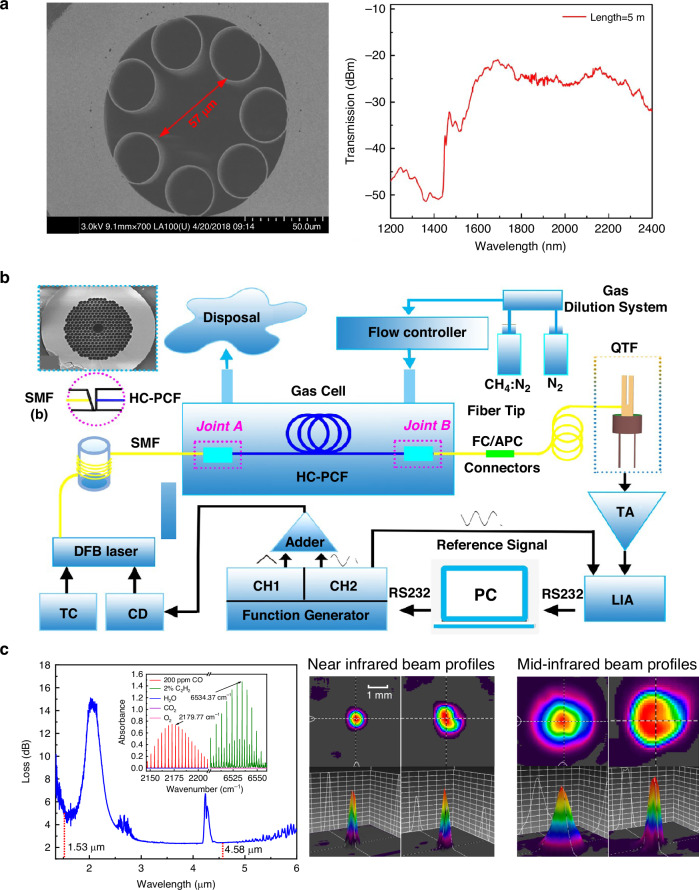
**Optical waveguide-based LITES sensor**. **a** Transmission spectrum and scanning electron microscope image of the cross-section of the used HC-ARF^119^. **b** Schematic diagram of compact all-fiber LITES sensor^120^. **c** Loss spectra of the used HWG and beam profiles of input and output of HWG^121^. **a**–**c** are reprinted from refs. ^119–121^, respectively, with permission from the Optica Publishing Group

Figure 23a shows the scanning electron microscope image of the HC-ARF’s cross-section. The fiber consists of a single layer of seven non-touching silica capillaries, featuring a distinctive hollow-core structure. This design not only increases the effective interaction length between light and gas but also maintains low transmission loss while effectively suppressing mode interference, thereby achieving high sensitivity in gas detection. Furthermore, Fig. 23b illustrates the transmission characteristics of the HC-ARF, showing efficient transmission across a broad spectral range from 1.45 to 2.4 μm. To address the challenges of coupling spatial laser beams into optical fibers, an all-fiber LITES sensor has been proposed. This design, based on an all-fiber structure, not only ensures efficient coupling of the laser beam but also enhances the system’s resistance to environmental interference. In 2020, a compact all-fiber LITES technique was proposed for trace gas detection, using a hollow-core photonic crystal fiber (HC-PCF)^120^. The schematic diagram of the sensor is shown in Fig. 23c. HC-PCF facilitates light transmission through periodically arranged air holes, providing excellent dispersion characteristics and flexibility. This fiber-based LITES sensor has the potential for long-distance and multi-point detection, opening up opportunities for integration into photonic circuits.

However, the diameter of HC-PCFs, typically on the order of hundreds of micrometers and significantly smaller than conventional fibers, presents challenges in efficiently injecting laser beams into the hollow core. The emerging hollow-core waveguide (HWG), with an inner diameter of approximately 4 mm, alleviates these challenges. It not only reduces optical losses caused by mode mismatch or fiber alignment issues but also simplifies overall system integration and operation. In 2023, Chen et al. proposed a LITES gas sensor based on the HWG^121^. The inner wall of the HWG is coated with a silver (Ag) film, which enhances light reflectivity and substantially increases the coupling efficiency of the optical waveguide. Additionally, the larger inner diameter of the HWG reduces the difficulty of optical coupling, and its broad transmission range provides an effective transmission channel for lasers of different wavelengths. In the experiment, a 65 cm long HWG with an inner diameter of 4 mm was used as both the light transmission medium and the gas cell. A DFB diode laser with an output wavelength of 1.53 µm and a QCL with an output wavelength of 4.58 µm were selected to target C₂H₂ and CO gases, respectively, to verify the performance of the HWG-based LITES sensor in both the near-infrared and mid-infrared regions. The laser beams maintained an ideal Gaussian distribution, confirming the advantages of the HWG in preserving beam quality and optimizing coupling efficiency. In addition, based on the same HWG, a CO-LITES sensor operating at 2.33 µm has also been reported^122^. In 2024, Chen et al. further conducted research on an all-fiber CO-LITES sensor operating in the 2.33 μm wavelength band, using the HC-ARF as the gas cell^123^. A comparison of gas detection performance using optical waveguides is presented in Table 6.

**Table 6:** Comparison of optical waveguide-based LITES sensors

Types of optical waveguides	OPL (cm)	Target gas	Gas absorption line (cm^−1^)	MDL @ Integration time
HC-ARF^119^	75	C_2_H_2_	6534.37	4.75 ppm @200 ms
		CO	6380.30	1704.00 ppm @200 ms
All-fiber HC-PCF^120^		CH_4_	6046.95	102.45 ppm @60 ms
Optical waveguide	65	C_2_H_2_	6534.37	6.07 ppm @200 ms^121^
		CO	2179.77	98.66 ppb @200 ms^121^
		CO	4288.26	31.60 ppm @200 ms^122^
All-fiber HC-ARF^123^	55	CO	4291.50	27.53 ppm @200 ms

In LITES systems, innovations in optical cavity design and waveguide technology directly drive the enhancement of detection performance. By employing structures such as Herriott MPCs, dense-spot MPCs, and integrated cavities, the number of light beam reflections is significantly increased, extending the optical-gas interaction path by 1–2 orders of magnitude. This substantially amplifies absorption signal strength and detection sensitivity. In contrast, the application of optical waveguides overcomes size limitations, simplifies optical alignment processes, and enhances both system integration level and mechanical robustness. These technologies collectively propel LITES toward higher sensitivity, smaller footprint, and stronger environmental adaptability, ultimately enabling breakthrough performance in trace gas detection on miniaturized platforms.

### Modified QTF-based LITES sensor

As the core detection component of LITES sensors, the performance of the QTF directly impacts the sensor’s detection limit. Therefore, improving the detection performance of the QTF is crucial for achieving lower detection limits. Optimization efforts have primarily focused on three key approaches: (1) Enhancing QTF performance by using custom low-frequency QTFs; (2) Boosting detection signal strength by coating the QTF with materials that increase thermal expansion or optoelectronic coupling effects; (3) Increasing the interaction length between the laser and the QTF. The enhancement of QEPAS sensor performance through the use of custom low-frequency QTFs, including the design of QTF dimensions and shape, is discussed in the section “QEPAS-based gas sensing .” This approach has also been effectively applied to enhance the performance of LITES sensors^116,117^.

#### Coated QTF-based LITES

**Fig. 24:**
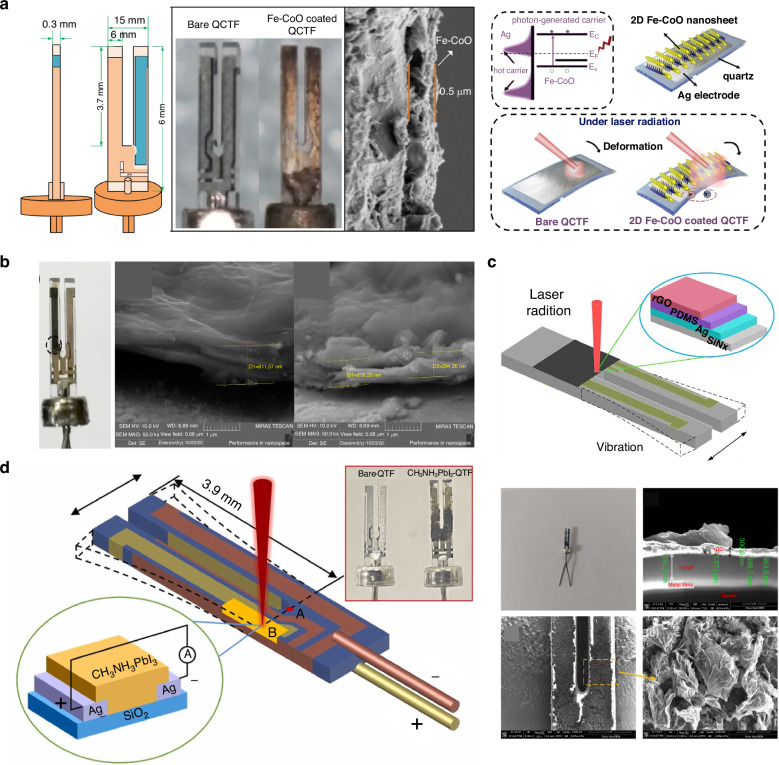
**Different types of QTFs for light intensity sensing**. **a** 2D Fe-CoO coated QTF^124^. **b** Graphene-enhanced QTF^125^. **c** Reduced graphene oxide/polydimethylsiloxane coated QTF^126^. **d** Photoelectric-thermoelastic co-coupling QTF^127^. **a** is reproduced from ref. ^124^ with permission from Elsevier. **b**, **c** are reproduced from ref. ^125^ and ref. ^126^, respectively, with permission from the IEEE. **d** is reproduced from ref. ^127^ with permission from Elsevier

Special materials can be coated onto the QTF to enhance its laser energy absorption and photoelectric conversion efficiency, without requiring any change in the size of the QTF. This approach offers the advantages of simplicity and low cost. In 2021, Zhou et al. proposed a LITES gas sensor based on a QTF coated with an ultrathin iron-doped cobalt oxide (Fe-CoO) two-dimensional (2D) film^124^, as shown in Fig. 24. The Fe-CoO film, with its large specific surface area and high charge mobility, significantly enhanced the light-induced thermal-elastic conversion efficiency of the QTF, resulting in a stronger piezoelectric signal. The sensor demonstrated highly sensitive detection of CH_4_, achieving a NNEA coefficient of 2.2 × 10^−10^ cm^−1^ W Hz^−1/2^. This study highlights the potential of developing new types of QTF detectors using ultrathin 2D materials, which offer an ultra-wide wavelength response range and high thermal-elastic conversion efficiency. In 2021, Lou et al. developed a graphene-coated QTF-based LITES gas sensing system^125^. This system improves light absorption and thermoelastic effects by depositing graphene thin films of varying thicknesses onto commercial QTFs. As shown in the SEM image of Fig. 24b, the thin films form a layered macrostructure with double-layer gaps, increasing the surface area and enhancing light absorption. It was observed that the QTF frequency decreased as the graphene thickness increased. CO_2_ was chosen as the target analyte for performance validation. Compared to the traditional LITES system using a bare QTF, the sensitivity and SNR were enhanced by 1.8-fold and 1.7-fold, respectively. This method of coating graphene onto commercial QTFs via spin-coating is low-cost, simple, and easy to operate, paving the way for the practical use of graphene-coated QTFs in trace gas analysis and ultra-wideband optical detection.

Building on these studies, in 2022, Wang et al. reported a novel LITES gas detection technology based on a QTF coated with polydimethylsiloxane (PDMS) and reduced graphene oxide (rGO)^126^, combining the advantages of both materials. The PDMS coating, with its excellent thermal expansion coefficient, significantly improved the thermoelastic conversion efficiency of the QTF. Meanwhile, the rGO coating, with its superior light absorption and thermal conductivity, further enhanced the sensor’s sensitivity. Details of the PDMS-rGO-coated QTF are shown in Fig. 24c. SEM images revealed that the coated region exhibited a layered, stacked macrostructure, which increased the surface area and promoted light absorption. Experimental results indicated that compared to the bare QTF, the introduction of the PDMS-rGO composite coating increased the signal amplitude and SNR by 8.64-fold and 5.53-fold, respectively.

In 2023, a perovskite-coated QTF-based LITES gas detection method was proposed^127^. The CH_3_NH_3_PbI_3_ perovskite thin film was deposited on the surface of a standard QTF, forming a Schottky junction with the silver electrodes on the QTF, as shown in Fig. 24d. The perovskite material, CH_3_NH_3_PbI_3_, is known for its high absorption coefficient and excellent photoelectric and thermoelastic properties. When the Schottky junction is formed on the QTF surface, it efficiently converts light energy into both electrical and thermal energy, significantly enhancing the signal response of the LITES system. Experimental results revealed that the perovskite-coated QTF increased the 2*f* signal amplitude and SNR for O_2_ detection by approximately 106-fold and 114-fold, respectively, compared to the bare QTF. A comparison of coated QTF-based LITES sensors is presented in Table 7.

**Table 7:** Comparison of coated QTF-based LITES sensors

Types of coated materials	Target gas	Gas absorption line (cm^−1^)	MDL @ Integration time	Performance improvement factor
Fe-CoO^124^	CH_4_	6046.58	0.88 ppm @1 s	4.50
Graphene^125^	CO_2_	6327.11	0.06% @100 ms	1.70
PDMS-rGO^126^	NH_3_	6612.70	46.01 ppm @100 ms	5.53
Perovskite^127^	O_2_	13142.58	260.00 ppm @10 ms	114.00

#### Side-excitation QTF-based LITES

**Fig. 25:**
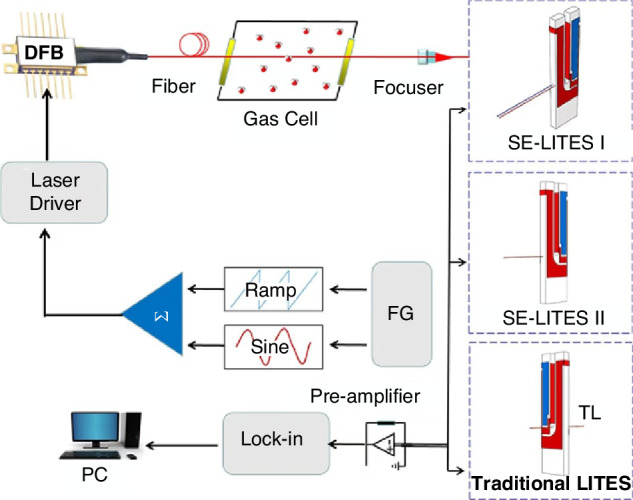
**Schematic of the SE-LITES sensor: SE-LITES I and SE-LITES II represent different laser excitation positions**^128^. The figure is reprinted from ref. ^128^ with permission from the Optica Publishing Group

In traditional LITES technology, the laser beam is typically focused on the central front surface of the QTF (as shown in Fig. 25), with the light absorption path limited to the thickness of the QTF. In 2023, Zheng et al. introduced a side-excitation LITES (SE-LITES) technique^128^, which enhances gas detection sensitivity by optimizing the focus and incidence angle of the laser, thereby increasing the interaction length between the laser and the quartz substrate. In SE-LITES, the laser is directed onto one side surface of the QTF, allowing the beam to penetrate the entire crystal. This significantly extends the light absorption path, which is now equivalent to the width of the QTF, thus improving laser energy absorption and enhancing the efficiency of thermoelastic conversion. In their research on SE-LITES, Zheng et al. selected a 32-kHz QTF and removed part of the silver electrode from its side surface, creating an electrode-free area through which the laser beam can enter and penetrate the QTF crystal. As shown in Fig. 25, the performance of the LITES sensor is evaluated at three different focal positions of the laser beam. When the laser is focused on the side of the QTF near the base of the fork fingers (SE-LITES I), this position has been optimized to yield the maximum signal response. SE-LITES I benefits from the higher mechanical stress and longer light absorption path on the side surface of the QTF, which leads to an enhanced thermoelastic effect. Compared to the traditional LITES technique, SE-LITES technology achieves more than a 22-fold increase in signal amplitude and a 15-fold improvement in SNR.

To break through the detection limit of LITES sensors, enhancing the performance of their core detection component, the QTF, is critical. Current optimization strategies primarily manifest in three major performance enhancement trends: First, directly enhancing its intrinsic performance through the customized design of low-frequency QTFs. This method has been validated in both QEPAS and LITES sensors, significantly improving fundamental sensitivity. Second, amplifying signal strength by coating the QTF surface with special materials, which effectively amplifies the detection signal by introducing external physical effects. Finally, improving energy coupling efficiency by extending the interaction length between the laser and the QTF further enhances the signal response. Collectively, customized QTF design provides a fundamental performance leap, coating technology achieves signal amplification, and increasing the interaction length optimizes energy utilization efficiency. The synergy of these three approaches drives LITES technology towards the goal of achieving lower detection limits.

### Heterodyne-based LITES

Currently, in the field of gas sensing, alongside high demands for detection sensitivity, there is also a growing requirement for system response speed. In quartz-enhanced laser spectroscopy sensing technology, where QTFs serve as detection elements, conventional techniques exclusively utilize the steady-state response of the QTF. Combined with harmonic demodulation technology, acquiring a complete signal waveform typically requires several tens of seconds. However, QTFs also have transient response characteristics. By implementing heterodyne demodulation mechanisms, the gas concentration can be retrieved through transient signals generated by the QTF, thereby enhancing system response speed. In heterodyne-based quartz-enhanced laser spectroscopy sensing technology, the system response time can achieve millisecond-level performance.

#### Typical heterodyne LITES

In 2021, Wu et al. proposed beat frequency QEPAS for fast^129^, calibration-free trace gas measurement, also known as H-QEPAS technology. This technique exploits the transient response characteristics of QTFs to enable rapid gas detection. In 2022, Wei et al. adapted this heterodyne approach to light-induced thermoelastic spectroscopy (H-LITES)^130^. By incorporating the heterodyne method, the H-LITES sensor facilitates rapid, highly sensitive, and accurate trace gas detection, while simultaneously providing information about the resonance characteristics of the QTF^131^. The demodulation of the QTF’s transient response signal at a non-resonant frequency, following absorption of a photothermal pulse, allows for the concurrent acquisition of the QTF’s resonant frequency, Q-factor, and gas concentration data. The H-LITES method is implemented by modulating the laser frequency to *f* = *f*_*0*_ + Δ*f*, where Δ*f* ≪ *f*_*0*_. A fast-pulsed ramp current is applied to the laser current driver, enabling rapid scanning across the targeted absorption line, while simultaneously introducing a sinusoidal modulation at frequency *f*. The resulting heterodyne response signal from the QTF is then demodulated at frequency *f* using a LIA. This process generates a beat frequency signal, which exhibits an exponentially decaying sinusoidal waveform, with frequencies between the QTF’s resonance frequency *f*_*0*_ and the laser modulation frequency *f*.

**Fig. 26:**
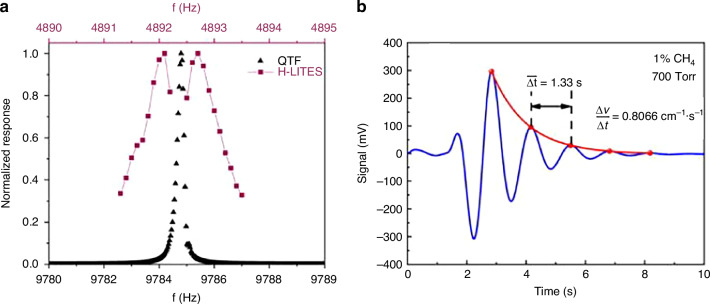
The parameter and detected signal of H-LITES sensor system. **a** H-LITES signal as a function of the excitation frequency^130^. **b** H-LITES as a function of time for a demodulation frequency of 9785.42 Hz (Δ*f* = 0.62 Hz). The red line is the envelope function of positive peak values^130^. **a**, **b** are reproduced from ref. ^130^ with permission of AIP Publishing

In 2021, a custom QTF with a fundamental resonant frequency of 9.78 kHz was employed^130^. As shown in Fig. 26a, the maximum H-LITES signal is obtained when the detuning frequency is Δ*f* = 0.62 Hz. Following the ramp excitation, the QTF begins to release the excess stored energy, vibrating at its fundamental mode. Figure 26b shows the H-LITES signal as a function of time with a detuning frequency of Δ*f* = 0.62 Hz. An oscillation period of Δ*t* = 1.33 s is observed, corresponding to a resonant frequency of *f*_*0*_ = 9784.95 Hz. The positive peak values for each oscillation are extracted and fitted to an exponential decay function *V*_*0*_·exp(−*t*/*τ*_*QTF*_), yielding *τ*_*QTF*_ = 1.18 s and *Q* = π*f*_*0*_·*τ*_*QTF*_ = 36274. Additionally, the H-LITES sensor demonstrates a proportional increase in peak signal with varying gas concentrations.

#### Self-correlated H-LITES

**Fig. 27:**
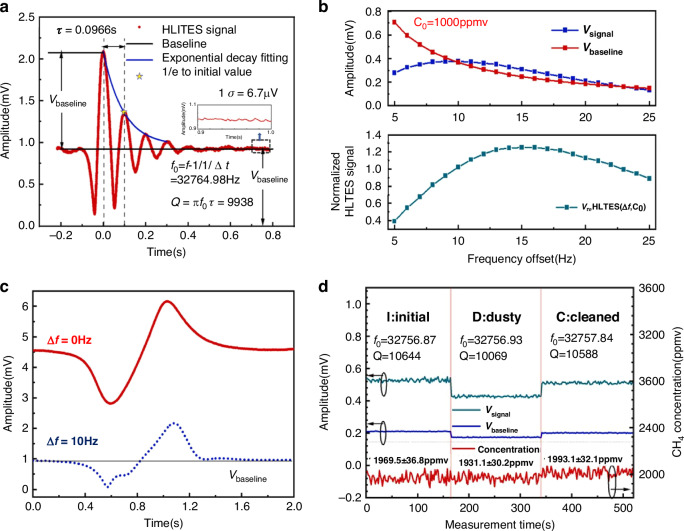
**SC-H-LITES-based CH**_4_ **detection**. **a** The measured time-domain H-LITES signal for a 1000 ppm CH_4_ sample^132^. **b** Curves of *V*_*signal*_, *V*_*baseline*_, and *V*_*n,H-LITES*_ versus frequency offset ∆*f*^132^. **c** The measured 1*f* LITES signals without ring-down with a frequency offset of 0 and 10 Hz^132^. **d** The measured *V*_*signal*_, *V*_*baseline*_, as well as the CH_4_ concentration with three different resonant states^132^. **a**–**d** are reproduced from ref. ^132^ with permission from Elsevier

In 2022, Ma et al. proposed a highly robust near-infrared CH_4_ sensor system based on self-correlated heterodyne light-induced thermoelastic spectroscopy (SC-H-LITES)^132^. This technology effectively reduces the influence of laser power variation, laser focus position shift, and QTF resonant frequency change on measurement results by exploring the correlations in the time-domain heterodyne response of the QTF. The SC-H-LITES technique normalizes the H-LITES signal with the baseline signal, yielding a normalized H-LITES signal *v*_*n,HLITES*_ (Δ*f*, *C*) that is proportional to the gas concentration *C* and independent of laser power and focus position. Furthermore, the correlation between the normalized H-LITES signal and frequency offset is utilized to compensate for measurement errors caused by QTF resonant frequency shifts. Figure 27a illustrates the H-LITES signals measured at a CH_4_ concentration of 1000 ppm, including key parameters such as signal amplitude, baseline noise. From these parameters, the values of *f*_*0*_ and Q-factor are calculated to be 32764.99 Hz and 9938, respectively, which closely match the results obtained by electrical measurement methods of 32765 Hz and 9982.

Figure 27b demonstrates the variations in *V*_*signal*_, *V*_*baseline*_, and *V*_*n*_, H-LITES at different frequency offsets Δ*f* with a 1000 ppm CH_4_ concentration. By correcting Δ*f*, the measurement errors caused by changes in the QTF’s resonant frequency can be compensated for. Figure 27d further illustrates the *V*_*signal*_, *V*_*baseline*_, and corrected CH_4_ concentrations measured using QTF in its initial state, after exposure to dust, and following dust removal. This shows that, even when the QTF parameters change, the SC-H-LITES technique can accurately measure CH_4_ concentrations. Therefore, the SC-H-LITES technique is comparable to other QTF-based sensors in terms of detection sensitivity, while its stability in practical applications is significantly enhanced due to the adoption of the self-correlation measurement correction scheme.

#### F-P cavity-based H-LITES

In 2023, a F-P cavity-based phase demodulation method was proposed for heterodyne light-induced thermoelastic spectroscopy (H-LITES)^133^. This approach detects the vibration of the QTF using an F-P cavity, thereby avoiding the thermal noise typically generated in traditional LITES by direct laser irradiation on the QTF. This results in improved SNR and enhanced measurement stability of the sensor system. In the study, an F-P cavity was constructed using the end face of a single-mode fiber and one prong of the QTF, enabling precise measurement of the QTF’s vibration. As the QTF vibrates, the length change of the F-P cavity induces phase shifts, and by demodulating these phase changes, heterodyne signals that are directly related to gas concentration can be accurately extracted. Experimental results show that, compared to traditional intensity demodulation methods, the phase demodulation technique based on the F-P cavity offers superior stability and consistency under varying laser power and wavelength conditions. It also demonstrates stronger immunity to fluctuations in the laser source and environmental interference.

**Fig. 28:**
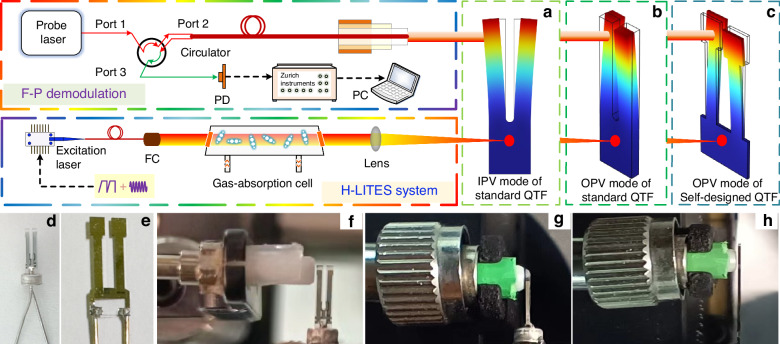
**The H-LITES sensor with F-P phase demodulation**. **a** H-LITES sensor-based IPV mode with standard QTF^134^. **b** H-LITES sensor based on OPV mode with standard QTF^134^. **c** H-LITES sensor based on OPV mode with self-designed QTF^134^. **d** The physical image of standard QTF^134^. **e** The physical image of self-designed QTF^134^. **f** The physical image of the traditional F-P cavity composed by standard QTF based on IPV mode^134^. **g** The physical image of the proposed F-P cavity composed by standard QTF based on OPV mode^134^. **h** The physical image of the proposed F-P cavity composed by self-designed QTF based on OPV mode^134^. **a**–**h** are reproduced from ref. ^134^ with permission from Elsevier

In 2024, Lang et al. further enhanced the sensitivity of H-LITES gas detection by utilizing the out-of-plane vibration (OPV) mode of the QTF^134^, as shown in Fig. 28. The vibration behavior of the QTF varies significantly depending on the vibration mode. In the traditional in-plane vibration (IPV) mode, illustrated in Fig. 28a, the tines of the QTF vibrate around the base of the fork. While this mode is commonly used, it has a higher resonant frequency, which limits the energy accumulation time of the system. In contrast, in this OPV mode, shown in Fig. 28b, the tines vibrate perpendicular to the base plane, which results in a lower resonant frequency. This enhances the system’s response to photothermal effects, prolongs the energy accumulation time, and ultimately improves sensor sensitivity. Under identical experimental conditions, the OPV-H-LITES sensor demonstrated an SNR 3.5 times higher than that of the IPV-H-LITES sensor when using a standard QTF. To further optimize sensor performance, a custom-designed low-frequency QTF, shown in Fig. 28c, was used to replace the standard QTF. This modification increases the SNR of the OPV-H-LITES sensor by an additional 2.3 times.

Regarding the development of H-LITES technology, its performance exhibits a significant trend of progressive optimization: The typical H-LITES system achieves rapid and highly sensitive simultaneous measurement of gas concentration and QTF parameters through heterodyne detection; SC-H-LITES substantially enhances robustness through signal self-correlation normalization and frequency offset correction, effectively suppressing the influence of laser power fluctuations, focus position shifts, and QTF resonant frequency drift, thereby ensuring concentration measurement accuracy under complex conditions; The F-P cavity phase demodulation H-LITES innovatively adopts an indirect demodulation scheme, inferring gas concentration by demodulating the vibration displacement of the QTF. This method breaks free from the limitations of the QTF’s vibration modes, providing a new technical solution for multicomponent gas detection. Overall, through innovations in detection mechanisms, H-LITES technology continuously strengthens its anti-interference capability and expands application scenarios while maintaining high sensitivity and rapid response, laying a solid foundation for practical applications.

### QEPAS-LITES combination

Despite the initial success of LITES technology in achieving high-sensitivity gas detection, there is still significant room for improvement in terms of energy utilization and detection performance. To address this challenge, a dual-spectral detection method that integrates QEPAS with LITES has been proposed. This approach aims to leverage the complementary advantages of both technologies, enhancing the energy efficiency of laser use and boosting the performance of gas detection. The implementation of this approach primarily involves two schemes: (1) A dual-spectral detection method based on multiple QTFs, where QTFs are used to detect photoacoustic and light-induced thermoelastic signals, respectively; (2) A dual-spectral detection method based on a single QTF, exciting both photoacoustic and light-induced thermoelastic effects on the same QTF. The execution of these two schemes is designed to enhance detection performance, thereby providing a more reliable technical approach for the accurate identification of trace gases.

#### Multiple QTFs-based QEPAS-LITES combination

In 2021, Hu et al. reported a novel gas detection technique that combines LITES and QEPAS, referred to as quartz-enhanced photoacoustic-photothermal spectroscopy or QEPAS-LITES^135^. This technique achieves high-sensitivity detection of trace gases by simultaneously measuring the generated photoacoustic and light-induced thermoelastic signals by two QTFs. Compared to the use of QEPAS or LITES alone, the QEPAS-LITES system significantly enhances the signal level by superimposing these two signals. H_2_O was selected as the analyte gas to validate the performance of the QEPAS-LITES sensor. The results demonstrated that the 2*f* signal of the QEPAS-LITES system was enhanced by 10.2 times and 1.1 times compared to the use of QEPAS or LITES alone, respectively. However, the detection performance of the QEPAS-LITES sensor can be degraded due to the mismatch of the resonant frequencies of the multiple QTFs. To address this issue, in 2022, Ma et al. proposed a method of matching QTF resonant frequency by adjusting temperature or pressure^136^.

**Fig. 29:**
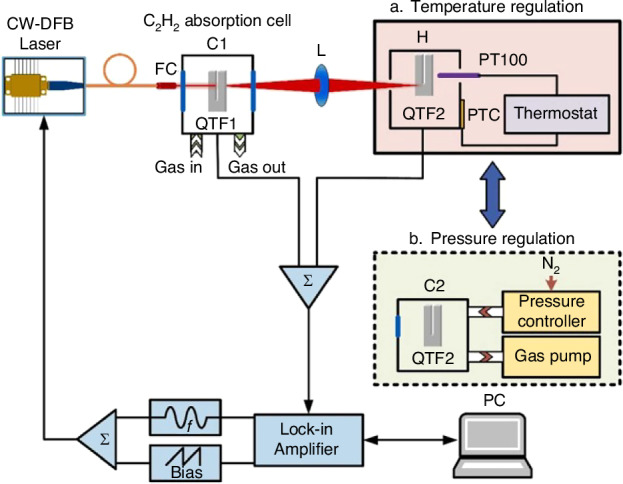
**Schematic diagrams of resonant frequency matching in QTFs for QEPAS-LITES based on temperature regulation and pressure regulation**^136^. The figure is reproduced from ref. ^136^ with permission from Elsevier

The resonant frequency of a QTF can be altered by adjusting the temperature and pressure, as the vibration characteristics of the QTF are directly affected by the surrounding environmental factors. The schematic diagrams of resonant frequency matching for QEPAS-LITES, based on temperature and pressure regulation, are shown in Fig. 29. By adjusting the temperature or pressure of QTF2 to match the resonant frequency of QTF1, effective signal superposition from the two sensors can be achieved. Experimental results demonstrate that, after adjusting the temperature, the superposition coefficient improves from 54.7% to 95.0% when the working temperature of QTF2 reaches 67.5 °C. Additionally, with pressure adjustment, the superposition coefficient further increases to 97.2% when the working pressure of QTF2 is set to 500 Torr.

#### Single QTF-based QEPAS-LITES combination

Although dual-spectral simultaneous detection can be achieved in the QEPAS-LITES system using multiple QTFs, this approach presents challenges such as difficulty in frequency matching between the QTFs, increased system complexity, and inefficient use of space, which limit the sensor’s practical applications. To address these limitations, a dual-spectral detection method based on a single QTF was proposed. This method improves the sensor’s performance by enabling the detection of both photoacoustic and light-induced thermoelastic signals with a single QTF. Not only does this simplify the system design, but it also enhances energy utilization and eliminates the need for frequency matching between multiple QTFs.

**Fig. 30:**
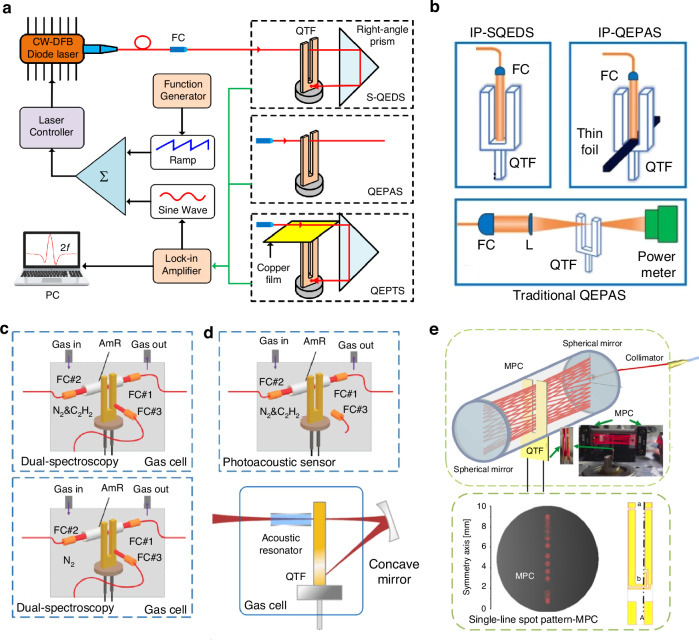
**Different implementation schemes combining QEPAS and LITES technologies**. **a** Schematic diagram of S-QEDS, QEPAS, and LITES sensors^137^. **b** Schematic diagram of IP-SQEDS, IP-QEPAS, and traditional QEPAS sensors^138^. **c** AmR and MPC-based dual-spectroscopy, QEPAS, and LITES sensors^139^. **d** Concave mirror-based QEPAS-LITES sensor^140^. **e** Folded-optics-based QEPAS-LITES sensor^141^. **a** is reprinted from ref. ^137^ with permission from the Optica Publishing Group. **b** is reproduced from ref. ^138^ with permission from MDPI. **c** is reproduced from ref. ^139^ with permission from the IEEE. **d**, **e** are reproduced from ref. ^140^ and ref. ^141^, respectively, with permission from Elsevier

In 2021, Qiao et al. introduced a novel trace gas sensor based on single-quartz-enhanced photoacoustic-photothermal dual-spectroscopy (S-QEDS) technology^137^. This approach utilizes a QTF to simultaneously detect photoacoustic and light-induced thermoelastic signals, which are then combined to enhance detection performance. The method not only improves detection sensitivity but also resolves the issue of signal amplification being affected by resonant frequency mismatches in multi-QTF systems. The configurations of the S-QEDS, QEPAS, and LITES sensor systems are illustrated in Fig. 30a. Experimental results showed that the 2*f* signal amplitude of the S-QEDS sensor was nearly equal to the sum of the individual amplitudes from the QEPAS and LITES sensors, demonstrating an ideal superposition effect. Building on the S-QEDS technique, Liang et al. proposed a gas detection method in 2022 based on in-plane single-quartz-enhanced dual spectroscopy (IP-SQEDS)^138^. As shown in Fig. 30b, this sensor enhances the effective interaction path between the laser and target gas molecules by directing the laser beam parallel to the QTF plane, where it then strikes the uncoated quartz at the bottom of the QTF, significantly boosting the signal amplitude.

In 2023, Bi et al. introduced a dual-spectroscopy gas detection sensor that utilizes an AmR to enhance QEPAS signals and an MPC to amplify LITES signals^139^. A partial structural diagram of the proposed sensor is shown in Fig. 30c. The laser beam is first emitted from FC#1 and passes through the AmR to generate the QEPAS signal. It is then received by FC#2, transmitted through the MPC for enhanced gas absorption, and delivered to FC#3 via an optical fiber, ultimately irradiating the prongs of the QTF to produce the LITES signal. The AmR plays a critical role in both enhancing the QEPAS signal and reducing the complexity of optical alignment through an off-beam path design. Additionally, the MPC effectively enhances the LITES signal, optimizing the use of laser energy.

Similar research was also conducted by Hu et al.^140^, as shown in Fig. 30d. In 2024, Cui et al. introduced a folded-optics-based quartz-enhanced photoacoustic and light-induced thermoelastic hybrid spectroscopy^141^, as illustrated in Fig. 30e. The MPC accommodates up to 60 laser beams within its cavity. Some beams partially strike the inner edge of the QTF, while others pass through the prongs of the QTF, thereby generating both photoacoustic and light-induced thermoelastic signals. In the sensor setup, the contributions of photoacoustic and light-induced thermoelastic effects from the multiple beams are approximately 7% and 93%, respectively. Compared with single QEPAS and LITES, the signal gain factors for such sensors were increased approximately 79 and 14 times, respectively.

The QEPAS-LITES combined technology significantly enhances gas sensing performance by synergistically measuring photoacoustic and light-induced thermoelastic signals. Its development has evolved along a clear trajectory: from multi-QTF setups towards single QTF integration, accompanied by reduced system complexity and continuous improvement in signal enhancement. Early multi-QTF schemes were constrained by resonant frequency mismatch between QTFs, necessitating additional temperature or pressure control for matching. To circumvent the matching challenges and spatial limitations inherent in multi-QTF systems, single QTF solutions emerged. These achieve a signal amplitude nearly equivalent to the sum of separate QEPAS and LITES signals, eliminate the need for frequency matching, and significantly simplify the system while improving energy efficiency. Subsequent technologies further optimized signals through structural innovations. Overall, by refining hardware integration and enhancing photonic-acoustic-thermal coupling efficiency, this technology continuously drives progress toward higher-level detection sensitivity.

## Prospects of quartz-enhanced laser spectroscopy technology

In the future, the development of quartz-enhanced laser spectroscopy sensing technology will extend far beyond merely pursuing higher sensitivity or smaller size. Its core strategic value lies in empowering ubiquitous, real-time, and intelligent gas sensing capabilities. Especially, in recent years, the rise and demand for machine learning integration, portable devices, and real-time multi-species detection have driven quartz-enhanced laser spectroscopic sensing technologies towards intelligent and integrated development. These emerging opportunities not only expand the application boundaries of quartz-enhanced spectroscopy techniques but also signify their immense potential in fields such as smart cities, precision medicine, and new energy. Among them, the integration of machine learning algorithms can further optimize signal processing and feature extraction capabilities, enabling precise material identification in complex backgrounds. The miniaturized design of portable devices, combined with low-power laser modules, makes on-site rapid detection possible, showcasing significant potential especially in environmental monitoring, industrial safety, and other domains. Real-time multi-species detection relies on high-speed spectral analysis and adaptive algorithms to simultaneously track multiple trace gases, providing new tools for atmospheric chemistry research and medical diagnosis. In the process of future application development, by focusing on addressing needs in fields such as environmental health, industrial safety, precision medicine, deep space exploration, and national security, and by actively expanding into emerging markets like personalized health, smart agriculture, smart cities, and hydrogen safety, quartz-enhanced laser spectroscopy sensing technology is poised to evolve from an advanced detection technique into a key platform technology underpinning the development of future intelligent societies.

However, quartz-enhanced Laser spectroscopy sensing technology still faces multiple practical challenges in its commercialization process and real-world implementation. Cost control is the primary obstacle, stemming mainly from the complex fabrication processes of high-performance QTFs and stable laser sources. Environmental adaptability presents a significant challenge, as temperature and humidity fluctuations, along with vibrational noise, can significantly impact the stability of the Q-factor of QTFs. The lack of standardization hinders industry adoption, as differing requirements for sensitivity and response speed across various application scenarios make it difficult to form a unified solution. On the commercialization path, breakthroughs are needed in miniaturized optical integration to reduce production costs, the development of adaptive compensation algorithms to address complex field environments, and the simultaneous establishment of domain-specific technical standard systems. Despite these challenges, the technology has demonstrated clear commercial value in high-frequency scenarios with rigid demands, such as industrial process monitoring and medical applications. With further advancements in science and technology, it may enter a critical breakthrough period in the future.

## Conclusions

This review summarizes recent advances in two QTF-based gas sensing technologies: QEPAS and LITES, with a focus on enhancing gas detection sensitivity, system stability, response speed, and structural properties. As the core of both technologies, the QTF detection element detects acoustic and thermoelastic signals, which differ from common photodetectors. This makes the two technologies insensitive to laser wavelength and capable of responding to signals generated by excitation from lasers of any wavelength. QEPAS and LITES each have their own advantages, with their performances being highly complementary. This paper introduces several state-of-the-art methods. For QEPAS sensors, the strategies to improve performance include: (i) using high-power lasers to excite more gas molecules for absorption; (ii) applying novel excitation sources with high absorption coefficients to strengthen the absorption; (iii) employing resonant cavities to amplify acoustic waves or adopting multi-pass structures to generate multiple acoustic wave sources; (iv) utilizing low-frequency QTFs to increase energy accumulation time. For LITES sensors, the strategies to improve performance include: (i) employing cavity enhancement techniques to boost absorbance; (ii) enhancing detection capabilities through QTF modifications or custom QTFs; (iii) leveraging the advantages of QEPAS technology to maximize laser energy utilization; (iv) utilizing the transient response characteristics of QTFs to build a heterodyne system to improve the response speed. The mentioned methods have proven to be effective and play a significant role in improving sensing performance.

The application of these technologies not only improves the detection performance of QEPAS and LITES-based sensors but also facilitates their practical applications. In the future, as cross-disciplinary convergence with various fields and technologies deepens, these two QTF-based spectroscopic technologies will evolve towards higher sensitivity, higher integration, even on-chip integration, and greater ease of practical application.
